# Small scale electrostatically-driven aerosol deposition in *airway-on-chip* models of bronchial constriction

**DOI:** 10.3389/fphys.2025.1621177

**Published:** 2025-09-16

**Authors:** Ron Bessler, Tirosh Mekler, Rami Fishler, Oshri Farhana, Sigal Dhatavkar, Tamar Daniel, Bar Kalifa, Kenichiro Koshiyama, Netanel Korin, Josué Sznitman

**Affiliations:** ^1^ Department of Biomedical Engineering, Technion – Israel Institute of Technology, Haifa, Israel; ^2^ Graduate School of Technology, Industrial and Social Sciences, Tokushima University, Tokushima, Japan

**Keywords:** airway-on-chip, pulmonary drug delivery, obstructive pulmonary disease, non-dimensional analysis, aerosol inhalation, electrostatics

## Abstract

Obstructive pulmonary diseases, including asthma and chronic obstructive pulmonary disease are widespread and represent a major global health burden. Despite their impact, effective therapeutic delivery to the small airways using inhaled aerosols remains suboptimal. In this study, we present a novel *in vitro* airway-on-chip platform that mimics both normal and constricted small bronchial geometries to quantify the deposition charged and neutral polystyrene latex aerosol particles ranging from 0.2 to 2 µm. Analytical and numerical solutions were derived from dimensionless scaling laws to further support the experiments and predict deposition location. Our experiments showcase how electrostatic forces significantly alter deposition patterns across particle sizes in these small airways. For submicron particles, we observe the enhancement of proximal airway deposition due to the coupling of electrostatic-diffusive screening effects. For larger particles, which typically deposit only in the direction of gravity, the inclusion of electrostatic forces significantly extends their deposition footprint, enabling deposition even in orientations where gravitational sedimentation is not feasible. Constricted regions consistently exhibit lower deposition across all cases, the presence of electrostatic forces enhanced overall deposition, offering a potential strategy for targeting bronchioles. Together, these findings suggest that electrostatic attraction may be strategically leveraged to enhance aerosol targeting in the small airways, providing new opportunities for optimizing inhaled drug delivery in obstructive lung diseases.

## Introduction

Obstructive pulmonary diseases, most notably asthma and chronic obstructive pulmonary disease (COPD), are highly prevalent across the globe and pose significant public health challenges. Despite the severity of the condition, effective treatment with inhaled aerosols remains limited, in part due to the poor deposition efficiencies of common inhalers, which typically deliver less than 50% of the inhaled dose (de Boer et al., 2017; Choi et al., 2019), with even lower efficiencies observed in pediatric populations (Oakes et al., 2023). The inefficiency in aerosol delivery is particularly pronounced in the presence of airway constriction, where the reduction in airway diameters creates higher resistance to airflow (Das et al., 2018; Oakes et al., 2023; Virchow et al., 2018). For example, in chronic bronchitis, a condition characterized by excessive mucus production and inflammation in the (small) bronchi, the accumulation of phlegm and swelling of the bronchial walls reduces the effective luminal space (Kim and Criner, 2013). Alternatively, the presence of mucus plugs occluding medium to large airways (i.e., approximately 2–10 mm lumen diameter) has been shown to be significantly associated with higher risk of all-cause mortality in COPD (Diaz et al., 2023).

In this landscape, delivering inhaled therapeutics to constricted airways, let alone obstructed ones, remains a vast challenge in pulmonary drug delivery. Recent inhalation therapy techniques have attempted to improve the outcomes of inhalation therapy, including modifying breathing patterns, using breath-actuated inhalers, and adjusting inspiratory flow rates (Ari and Fink, 2020). Nevertheless, the most widely used strategy remains optimizing the aerodynamic diameter (*d*_p_) of inhaled particles, as this parameter largely governs key deposition mechanisms such as impaction, gravitational sedimentation, and Brownian diffusion (Ijsebaert et al., 2001). Aerosol design criteria advocate that aerosols in the size range between approximately 2 and 6 μm hold the best potential to deposit in the central and small airways (Darquenne, 2012) as larger particles (>6 μm) tend to mainly deposit in the upper airways due to impaction whereas aerosols <2 μm deposit mainly in the deep lungs (i.e., alveolar regions). Despite such general guidelines, one significant particle property that is often overlooked in addressing aerosol deposition (Bessler and Sznitman, 2024) is the electric charge (*q*) acquired by aerosols during inhaler-generated formation (Kwok and Chan, 2009).

Briefly, when an inhaled charged particle approaches the (neutral) lung tissue, it induces a localized electric field triggering a dielectric effect (Wilson, 1947; Finlay, 2021; Balachandran et al., 1997). This effect causes surrounding charges or dipole molecules within the tissue to reorient in response to the particle’s projected field (*E*). The interaction between a particle’s inherent charge *q* and the induced dipoles or charges within the tissue results in an electrostatic attraction force *F*_*e*_; a phenomenon shown to enhance particle deposition in proportion to ${\left(Bq^{2}\right)}^{1/3}$ , where *B* is the particles mechanical mobility (Melandri et al., 1983; Cohen et al., 1998). While factors such as the carrier fluid (i.e., air), the gravitational field, particle density (which typically does not deviate significantly from that of water), and particle shape (often approximated as spherical, most notably for nebulized suspensions), are often challenging to modify significantly, the charge acquired by an aerosol can be engineered and customized (Kwok and Chan, 2009; Wilson, 1947). Inhalers can generate charged particles from near-neutral values following a Boltzmann distribution to the Rayleigh limit (Hinds and Zhu, 2022) (i.e., scenarios in which electrostatic repulsion overcomes surface tension of the droplet). For instance, a *d*_*p*_ *=* 1 μm droplet can carry a charge ranging from *q* ∼ 1 e to approximately 44,000 e. In fact, the ability to manipulate electrostatic charge is well established across the broader aerosol industry (Hinds and Zhu, 2022; Osman et al., 2015) and thus warrants further investigation in the context of pulmonary drug delivery (Bessler and Sznitman, 2024; Bailey et al., 1998).

To date, numerical simulations (Koullapis et al., 2016), *in vitro* studies (Bessler et al., 2023) and non-dimensional analysis (Bessler and Sznitman, 2024; Finlay, 2001) have underlined the importance of electrostatic charge towards deposition outcomes. Notably, these studies account for proper scaling based on realistic charge levels and particle sizes generated by commercial inhalers (Kwok and Chan, 2008). Electrostatic forces may play a dominant role in the deeper lung regions where characteristic length scales are small (Bailey et al., 1998; Finlay, 2001). There, electrostatic attraction, which is inversely proportional to the square of the distance between an aerosol and the luminal wall, can potentially overshadow conventional deposition mechanisms when particle charge is sufficiently elevated relative to size (Bessler and Sznitman, 2024; Bessler et al., 2023; Finlay, 2001). Yet, studies addressing the induced electrostatic forces have largely focused on deposition in large airways (Cohen et al., 1995; Xi et al., 2014). This observation results amongst others from historical and technical challenges of exploring the phenomenon at true scale in small bronchioles. Concurrently, while electrostatic forces are expected to play a significant role in the smaller lung regions, electrostatic attraction obeys the principle of superposition in addition to other deposition mechanisms and thus also contributes to deposition in the extra-thoracic and upper airways. Despite some recent interest in the field (Bessler et al., 2023), there remains a dearth of available data exploring electrostatic-driven pulmonary deposition at small scale (Sznitman, 2022).

Motivated by the ongoing shortcomings on aerosol electrostatics in the lungs, we investigate *in vitro* the role of electrostatic forces on aerosol deposition within small airway models. Specifically, we focus on how electrostatic forces influence deposition patterns in normal and constricted bronchioles. To this end, we present a novel *in vitro* airway-on-chip lined with electrically conductive material to mimic the conductive properties of the luminal airway tissue. We capture the size and branching structure of small to terminal bronchioles (i.e., corresponding to generations 12–15 of the seminal Weibel A model (Weibel, 1963)); the primary sites of airflow obstruction in COPD that contributes to the disease’s characteristic symptoms (Xu et al., 2022). We quantify aerosol deposition patterns spanning aerosol sizes of 0.2 µm–2 μm, representing particles typically influenced by Brownian diffusion and gravitational sedimentation, respectively (Hofem et al., 2015). Our experiments examine two specific charge distributions: (i) intrinsically charged atomized particles (>100 e) and (ii) neutralized particles following a Boltzmann distribution. By directly comparing these distributions, in conjunction with particle size, we attempt to shed new quantitative light on how electrostatic charge can overcome traditional deposition mechanisms and ultimately alter local deposition outcomes.

## Methods

### Aerosol exposure experiment

#### Aerosol exposure assay

**FIGURE 1:**
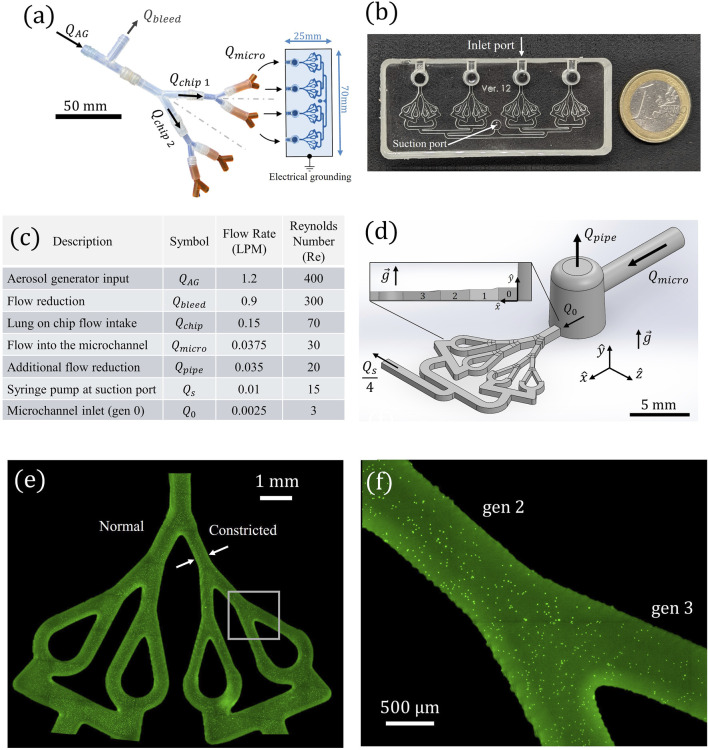
**(a)** Schematic of the experimental aerosol exposure setup. The air reduction apparatus expels excess airflow $\left(Q_{bleed}\right)$ from the original aerosol generator airflow output ( $Q_{AG}$ ), ensuring precise symmetrical flow distribution into the microchannels’ inlet port ( $\left.Q_{micro}\right)$ . **(b)** Photograph of the airway-on-chip device featuring four independent airway trees, with four inlets and a single outlet (suction port) where the syringe pump is connected. **(c)** Tabulated values of flow rate ( Q ) and it associated Reynolds number (Re) for each stage of the reduction process, showing the progressive decrease in airflow until reaching the inlet flow rate ( $Q_{0}$ ) into the individual airway trees. **(d)** Computer Aided Design (CAD) model of the microchannel system, illustrating the vertical pipe that reduces the final airflow reaching the individual airway tree showcasing three generations of bifurcating airway branches. The model is oriented such that the conductive flat plane is positioned against the direction of gravity to explore electrostatic effects counteracting gravitational forces. Subfigure presents side view to present the different lung generations: 0–3. **(e)** Fluorescent microscopy image showcasing deposition following an exposure experiment; **(f)** Inset: magnification of the fluorescent microscopy image of deposited individual particles in local airway branches. The case presented is for charged PSL particles with *d*_*p*_ = 0.5 μm.

The aerosols are generated using a collision-type atomizer (Model 3076, TSI) with filtered air as the gas source, maintained at P=2 bars. To minimize water vapor content while preventing particle loss, the aerosol stream is directed through two consecutive diffusion dryers (Model 3062, TSI). The resulting airflow rate is $Q_{AG}=$ 1.2 L/min (Figure 1a), measured using a flow meter (Model 4100, TSI). Such airflow rate is about three orders of magnitude higher than the desired bronchiolar flow rate (*Q*_0_) under quiet breathing conditions (Pedley, 1977) to feed the first generation of the four microchannel models positioned on a single microscope slide (Figures 1a,b). To achieve the required flow conditions, a stepwise air reduction and splitting mechanism is implemented, as detailed in the table of Figure 1c. The first stage of flow reduction is achieved using an assembled array of tubes (all apparatus dimensions are provided in the Supplementary Material (SM) Supplementary Figure S1a and S1b), which gradually reduces the airflow in a manner analogous to airway branching in the lungs, from the bronchi down to the bronchioles. This system, referred to as the “air reducer” (Figure 1a), expels approximately ∼80% of the excess airflow (*Q*_bleed_) through a 90° outlet into a chemical hood. The remaining airflow is symmetrically split to feed two parallel lung-on-chip models (*Q*_chip1_ and *Q*_chip2_). Each of these flows supplies four microchannels (*Q*_micro_) through their respective inlet ports (Figure 1b).

**FIGURE 2:**
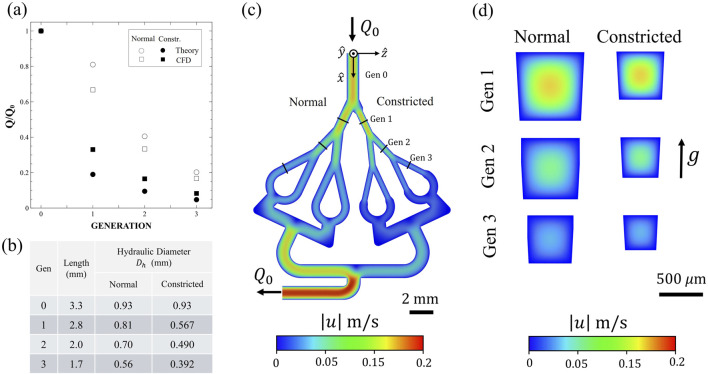
**(a)** Comparison between the theoretically (circular symbols) expected flow rate (based upon laminar Hagen-Poiseuille flow) to our Computational Fluid Dynamics (CFD) predictions (square symbols) at each airway generation. Comparison between the two airway conditions also presented: normal (empty symbols) and constricted (filled symbols) models. The values are normalized relative to that at Gen. 0. **(b)** Table summarizing airway dimensions, including generation length *L* and hydraulic diameter $D_{h}$ . **(c)** Top view of the numerically obtained velocity magnitude (log-scale) at the center plane of the full model. **(d)** Corresponding cross-sectional velocity distributions of the axial flow across different airway generations.

Before entering the first microchannel generation (i.e., Gen. 0, see Figure 1d and top view in Figure 2c) the bulk airflow passes through a small vertical pipe for additional reduction *Q*_pipe_. The desired flow rate into Gen 0, .designated as *Q*_0_, is then drawn under a constant steady flow into the microchannels *via* a syringe pump (PHD Ultra, Harvard Apparatus) connected to the suction outlet of each chip (Figure 1b; Supplementary Figure S1a). The constant flow approximation in the model is sufficient to accurately represent physiological breathing, as further justified in the device design section. The final reduction step ensures that *Q*_0_ is only 6% of *Q*_micro_ and is directed to the model inlet to reach the desired quite breathing rate, while the remaining 94% is expelled. The syringe pump simultaneously withdraws a total airflow of *Q*_*S*_ = 4*Q*_0_. Within each microchannel, the airflow is further subdivided in a manner mimicking bronchiolar bifurcation. The airway-on-chip design features individual airway trees with three bifurcating generations, includes both normal and constricted segments.

#### Fluorescent particle aerosolization

Fluorescent polystyrene latex (PSL) particles are used to investigate size-dependent aerosol deposition (*ρ*_*p*_ = 1,050 kg/m^3^, Fluoromax red and green, fluorescent microspheres, 1% solid, Thermo Scientific). The use of PSL particles is well established in aerosol exposure studies involving airway-on-chip models (Bessler et al., 2023; Elias-Kirma et al., 2020; Fishler et al., 2015) due to their well-characterized physiochemical properties and charge acquisition during aerosolization. To examine the effects of particle size and electrostatic charge on deposition, distinct monodisperse PSL sizes are used: $d_{p}$ = 0.2, 0.5 and 2 μm. While the smallest particles (i.e. 0.2 μm) are known to be significantly affected by Brownian diffusion, the largest ones (i.e. 2 μm) are subject to considerable sedimentation in small airways. In contrast, 0.5 μm particles sit in a size range where airborne transport is primarily driven by convection such that aerosols are exhaled under normal breathing and deposit in minimal amounts due to weak diffusive or gravitational forces (Hofem et al., 2015; Sznitman, 2013). Furthermore, and most relevant to the present study, PSL particles are selected due to their natural tendency to acquire electrostatic charge during the aerosolization process. The “Charged Group” consists of particles exiting the aerosol generator after diffusion drying, with an estimated mean charge of >100 e based on previous experimental data (Forsyth et al., 1998; Whitby and Liu, 1968). In contrast, the “Neutralized Control Group” is obtained by employing an electrical ionizer (Model 1090 MSP, TSI) and ensuring charge neutralization following a Boltzmann charge equilibrium. Under these conditions, the mean charge per particle is expected to be *q* ∼ 1 e for 0.2 μm and *q* ∼ 3 e for 2 μm particles (Hinds and Zhu, 2022). Note that these levels are insufficient to induce significant electrostatic deposition, allowing for direct comparison between charged and neutralized aerosols (Osman et al., 2015).

Prior to exposure (see previous section), polystyrene latex (PSL) particles are suspended in deionized (DI) water at a high concentration (∼10^7^–10^8^ particles/mL) to ensure sufficient deposition data for ensemble statistics within reasonable exposure times (∼30–90 min, depending on particle size and charge conditions) (Fishler et al., 2015). The particle suspension is prepared at a weight concentration of 5%, carefully selected to maintain an aerosol with <1% aggregate formation. This assumption follows from the expected evaporation of water droplets exiting the atomizer, characterized by a count mean diameter of 0.35 μm and a geometric standard deviation of 2 μm, as specified by the manufacturer. The size distribution of the generated aerosol is assumed to follow a log-normal distribution with monodisperse spherical particles, consistent with previous studies (Fishler et al., 2015; Raabe, 1968). To minimize aerosol aggregation due to the high particle concentration, each suspension undergoes 30 min of sonication in an ultrasonic water bath (Elma Elmasonic S10) immediately before experimentation. To prevent particle sedimentation during experiments, a magnetic stirrer continuously operates inside the suspension container.

#### Deposition quantification

Following each aerosol exposure experiment, the bronchiole microchannels were examined using an inverted fluorescent microscope (Nikon Eclipse Ti) at varying magnifications (×2, ×10 and ×20) depending on particle size (i.e., 0.2 μm, 0.5 μm and 2 μm respectively), see Figures 1e,f. Using ANDOR Zyla sCMOS camera, we captured the entire model by stitching them together with zero overlap, using large free-shape images function. To reduce the image memory space, we used 4 × 4 binning. The quantification of deposition involved identifying the precise planar locations (*x, z*) of individual deposited fluorescent particles through digital processing of local intensity maxima using ImageJ software. Subsequently, the particles were counted with correspondence to their two-dimensional (2D) location and the sum of deposited particles were obtained for each airway generation. The deposition fraction was then calculated as the percentage of deposited particles in a specific airway generation out of the total number of deposited particles across the entire model. This approach enables us to determine the spatial distribution of particle deposition within the *in vitro* model.

### Airway-on-chip

#### Device design

The *in vitro* airway-on-chip platform consists of four planar, symmetric airway path trees spanning four generations 0–3, as schematically shown in Figures 1, 2c. The tree design broadly mimics the deep bronchial airway branches (i.e., bronchioles) in the distal region of the conducting zone, with hydraulic diameters of <1 mm (Figures 1a,b). Each airway segment consists of square cross-sections, with dimensions summarized in Figure 2b. The chosen generation dimensions were based on morphometric measurements representative of typical length and diameter of an adult human lung, corresponding to generations 12–15, following the seminal works of Weibel (1963), Horsfield et al. (1971). These airway dimensions mimic the deep bronchial regions, referring to airways with a diameter smaller than 2 mm, which are associated with constricted diseases (McNulty et al., 2014). While the Weibel A model assumes idealized cylindrical airways, actual distal bronchiolar cross-sections in the human lung are often irregular and deviate from perfect circles (Hogg, 2004; Wang et al., 2020)*.* In our *airway-on-chip* design, we employed square cross-sections (Figures 2c,d) as a deliberate engineering choice to enable precise microfabrication (Figure 1b), optical access for high-resolution imaging (Figures 1e,f), and consistent channel alignment across multiple generations. Importantly, flow behavior and particle transport in rectangular microchannels remain comparable to those in circular channels when the hydraulic diameter (defined as *D*_ℎ_ = 4*A*/*P*, where *A* is the cross-sectional area and *P* is the wetted perimeter) and flow conditions (Re) are matched. To ensure physiologically relevant flow, we designed each generation as a square channel to have a hydraulic diameter *D*_ℎ_ equal to that of the corresponding cylindrical segment in the Weibel model (see Figure 2b). While minor deviations in flow may occur near corners due to reduced velocities, these are localized and do not significantly affect the overall transport and deposition behavior of aerosols. Therefore, the underlying physics remain valid despite the geometric simplification. The first airway (Gen. 0, see Figure 1d and see top view Figure 2c) serves as the model’s inlet, ensuring fully-developed flow conditions over a distance exceeding the anticipated entrance length *L*_*e*_ by more than two orders of magnitude at such low Reynolds numbers ( $L_{e}∼0.06\text{Re}D_{0}$ ). These conditions result in fully-developed laminar Poiseuille flow profiles within the model’s bronchioles (see Figures 2c,d).

Approximating a constant inhalation condition may be assumed for modeling airflow in the deep lung regions during quiet breathing, as the Womersley number (Wo = *fL* ^*2*^ *ρ*_*f*_ */μ*_*f*_), which is a dimensionless expression of the oscillatory airflow frequency (*f ∼ 0.25* Hz for quite breathing*)* in relation to viscous effects, remains well below unity in our bronchial region of interest (Wo ≪ 1), indicating quasi-steady flow conditions that can be approximated as steady flow conditions (Ménache et al., 2008). However, it is important to note that while the airflow can be considered steady, the absence of exhalation in the model may lead to overestimating particle retention, as particles that would otherwise exit the lungs during exhalation are not accounted for. The Gen. 0 segment terminates in a bifurcation with a 25° split angle. Beyond this point, the airway tree bifurcates symmetrically across all generations, maintaining a consistent 37° branching angle along the symmetry line (Ménache et al., 2008). The model branches into two distinct pathways: one representing normal anatomical bronchioles and the other featuring a 30% constriction, designed to mimic smaller airways or regions affected by obstructive pulmonary diseases (Figure 2). Airflow regulation is achieved using a syringe pump connected to the model outlet port (Figure 1b). The present design enables the investigation of electrostatic deposition by incorporating a conductive coating, while electrical grounding prevents charge buildup (Bessler et al., 2023).

#### Device fabrication

Models were fabricated using 3D-printed molds printed using a PRUSA SL1 3D printer and 3DM-ABS Orange Tough Resin), see Supplementary Material Supplementary Figures S1c and S1d. The models were filled with polydimethylsiloxane (PDMS), inspired by recent microfluidic lung airway models (Bessler et al., 2023; Elias-Kirma et al., 2020). PDMS, a well accepted material for microfluidic devices, was selected for its optical transparency and high flexibility, allowing for easy molding into complex shapes and structures. The printing settings included a layer height of 25 μm and an exposure time of 10 s. Post printing, the molds were cleaned with isopropyl alcohol, and to solidify the resin the molds were cured at 60 °C for 1 h under ultraviolet light (Formlabs Form Cure FH-CU-01 curer). After pouring the PDMS, the PDMS-filled molds were placed in desiccators for 1 hour to release trapped air bubbles. The PDMS models were peeled after 24 h of resting within the mold under ambient conditions. The outlet port for the syringe insertion (Figure 1b) was created using a 2 mm biopsy punch (Miltex, 3331).

#### Conductive Layering

The electrical conductivity of lung parenchymal tissue closely approximates that of saline water $\sigma_{H_{2}O}∼1$ S/m (Osman et al., 2015). This high conductivity allows electrical charges within lung tissue to redistribute rapidly, facilitating the manifestation of image charge phenomena (Melandri et al., 1983). In contrast, glass has a significantly lower conductivity ( $\sigma_{\text{glass}}∼$ 10^−11^ S/m), which necessitates an alternative approach to achieve a physiologically relevant conductive environment.

To address this limitation, we applied a conductive indium tin oxide (ITO) coating to our microscope slide, following previous methodologies (Bessler et al., 2023). The ITO pattern was selectively deposited only in regions corresponding to the airway microchannels (see Supplementary Figure S2) to avoid interfering with the adhesion of the PDMS to the glass. The bonding process was performed *via* a 1 min plasma treatment (ETP, INC. Model BD-20). The ITO coating, provided by Huizhou Konshen Glass Co., Ltd., had a sheet resistivity of approximately $R_{s}∼10\;\Omega /m^{2}$ , significantly enhancing the surface conductivity to $\sigma_{\text{ITO}}$ ∼10^2^ S/m (see Supplementary Material Supplementary Table S1 for details on sheet resistivity). This elevated conductivity enables the manifestation of image charge effects, more faithfully replicating the electrostatic interactions expected in lung tissue. Notably, the electrostatic relaxation time scale ( $\tau_{e}∼1$ pico s) in real lung tissue is orders of magnitude shorter than the characteristic timescale of a particle ( $\tau_{p}∼\;1$ μs). Thus, achieving a sufficiently conductive environment in our model mimics more faithfully electrostatic behavior. Additional details on the relevant timescales are provided in the Supplementary Material (see Supplementary Material Conductive Layering of the Microchannel). To maintain a consistent electrical setup across experiments and prevent excess charge buildup during exposure assays, the coated conductive glass was electrically grounded using a custom external connector wired to the inner surface of the microfluidic channel (See Supplementary Figure S1a).

### Computational fluid dynamics (CFD)

Numerical simulations (see Figure 2; Supplementary Figure S3b) were performed using the commercial solver Fluent 24.2 (ANSYS, Inc.), employing a steady-state laminar flow model with air as the working fluid ( ρ = 1.22 kg/m^3^, μ = 1.78 × 10^−5^ Pa·s). The model’s boundary conditions included a zero-pressure inlet, an outlet flow rate of 2.5 mL/min, and a no-slip condition on the walls. The momentum equations were discretized using a second-order upwind scheme for velocity and a second-order scheme for pressure. Velocity-pressure coupling was handled *via* the SIMPLE algorithm, with a least-squares-based scheme for gradient calculations. The computational domain was meshed using Ansys Meshing, employing polyhedral cells ranging from 400K to 3M as part of a mesh convergence study.

### Analytical and numerical modeling

To gain a deeper understanding of particle deposition within the model, we explore the dynamics of aerosol transport and apply Newton’s second law to charged particles and derive their expected trajectory within the mid-channel *xy* plane (see Figure 1d; Supplementary Figure S3b). The governing forces considered in this analysis include (i) electrostatic attraction due to induced charge (−*k*_e_ *q* ^2^/4*y* ^2^), where *k*_*e*_ is the Coulomb constant, (ii) gravitational sedimentation (+*mg*), (iii) viscous Stokes drag (−*v*_rel_/*B*), where *v*_rel_ is the relative velocity of the particle with respect to the surrounding streamwise flow velocity *u*_*f*_ (whose profile varies along the *y*-direction) and *B* is the aerosol mechanical mobility, and (iv) Brownian motion (*F*_*B*_). Assuming no hygroscopic growth (i.e., constant particle mass *m*), the particle dynamics are described by the coupled equations of motion 1 and 2, presented below:

$$\hat{y}:\;mg-\frac{k_{e}q^{2}}{{4y}^{2}}-\frac{\dot{y}}{B}+F_{B_{x}}=m\ddot{y}$$ (1)

$$\hat{x}:\;-\frac{1}{B}\left(u_{f}\left(y\right)-\dot{x}\right)+F_{B_{y}}=m\ddot{x}$$ (2)

These equations capture the interplay between electrostatic, drag, gravitational, and diffusive transport mechanisms, providing a framework for predicting particle trajectories within the airway-on-chip model. Note that due to the electrostatic term (∝ *y* ^−2^), the governing equation in the *y*-axis (Equation 1) is a nonlinear, second-order, non-homogeneous differential equation, such that a direct analytical solution $y\left(t\right)$ for the particle height as a function of time is not feasible. Consequently, we extract particle trajectories over time and space using two approaches: (i) an Explicit Euler method, and (ii) non-dimensional analysis.

#### Explicit Euler method

We propose a canonical model featuring rectangular channels connected in series according to Weibel dimensions mimicking our *in vitro* model (Supplementary Figure S5). In each channel, the streamwise flow (*u*_*f*_) is approximated using the laminar Hagen–Poiseuille velocity profile (see Supplementary Figures S6 and S7, and derivations for flow in a square cross-section chanel) at low Reynolds number (Re ∼ 3), corresponding approximately to quiet breathing of a healthy person at these deep lung generations. The numerical simulations are initialized with an aerosol starting at a vertical distance of height $y_{\left(t=0\right)}=0.025D_{0}$ , chosen to align with the image charge method ( $\left.y\ll D_{0}\right)$ . Additionally, this distance from the tissue corresponds to approximately 10% of the annular volume in an ideal cylindrical airway (see Supplementary Figure S8 and derivation in the Supplementary Material), making it a widely accepted threshold for assessing whether electrostatic deposition is significant, as supported by previous studies (Finlay, 2021). This value will later be chosen as the characteristic length (*L*_*c*_ *= y*_*0*_ = 0.025*D*). The initial velocity is set based on the corresponding position in the Hagen–Poiseuille flow profile. Following each iteration, based on the time interval *Δt*, a 1D random Brownian step ( $\left.\pm \;\sqrt{2D_{diff}\Delta t}\right)$ was added to each calculated location, for each axis separately. Here, *D*_*diff*_ represents the Stokes–Einstein diffusion coefficient. To ensure convergence, the time step was set to 0.1 ms (*Δt* = 0.1 ms). To validate this choice, we performed simulations with progressively smaller time steps, ranging from 10 ms down to 1 μs, confirming that the solution converges (see Supplementary Figure S8a). Additionally, to account for the inherent error in the Explicit Euler method, where terms of O (*Δt* ^2^) and higher are neglected, we tracked the local truncation error (LTE) at each iteration. The resulting global truncation errors (GTE) remained around single microns and are summarized in the Supplementary Material (Supplementary Figure S8b; Supplementary Table S3). Additionally, to validate our numerical approach, we compared the results with an analytical solution derived from scaling laws. As the time step (*Δt*) decreases, both solutions converge well (see Supplementary Material Supplementary Figure S8 for comparison). Since our time intervals are much larger compared to the examined particle relaxation time (*τ*_*p*_ ≪*Δt*), the particle’s velocity instantaneously follows the surrounding flow velocity ( $\dot{x}=u_{f}$ ).

#### Non-dimensional analysis

Non-dimensional analysis is helpful for understanding aerosol transport and its leading physical mechanisms, in particular when electrostatic forces are involved. This approach expresses the dominant mechanisms in terms of characteristic length (*L*_*c*_ *∼ D*), velocity (*U*_*c*_ *∼Q/A*) and time (*t*_*c*_ *∼ L*_*c*_ */U*_*c*_) scales relevant to the bronchioles. We revisit the governing mechanisms of aerosol deposition in our model using dimensionless parameters (Sznitman, 2022; Tsuda et al., 2013). The deposition of small (approximately <0.5 µm) particles in the bronchioles occurs mainly *via* Brownian motion, characterized by the inverse Péclet number: Pe^*−1*^ *= D*_*diff*_ */U*_*c*_ *L*_*c*_. For larger particles (>1 µm), bronchiolar deposition is primarily governed by gravitational sedimentation, quantified by the gravity number H *= v*_*ter*_ */U*_*c*_, where *v*_*ter*_ is the settling velocity given by *v*_*ter*_ = *τ*_*p*_ *g*, and *τ*_*p*_ represents the particle relaxation time. Concurrently, particle inertia is captured by the particle Stokes number: Stk = *τ*_*p*_ *U*_*c*_ */L*_*c*_.

We then extend the discussion to when electrostatic charge is present. When Coulomb forces arising from particle–wall electrostatic interactions introduce an additional dimensionless group relevant to represent the Induced Charge *versus* the airflow convection (Finlay, 2001): Inc = (*Bk*_*e*_ */U*_*c*_ *) (q/L*_*c*_)^2^. By applying dimensional analysis to Newton’s second law, while omitting the noise introduced by Brownian motion (which can be superimposed subsequently), we obtain the non-dimensional governing equations for particle dynamics, Equations 3, 4, presented below:

$$\hat{y}:\;Hg^{\prime }-\text{Inc}-{\dot{y}}^{\prime }=\text{Stk }{\ddot{y}}^{\prime }$$ (3)

$$\hat{x}:\;-{\dot{x}}_{rel}^{\prime }=\text{Stk }{\ddot{x}}^{\prime }$$ (4)

where ${\dot{x}}_{rel}^{\prime }$ is the particle’s normalized horizontal velocity relative to the flow. Non-dimensional analysis in the deep bronchioles reveals that impaction is negligible (Stk ≪ Inc, H), and primarily occurs in the upper lung regions (Stahlhofen et al., 1989). As a result, the governing Equations 3 and 4, can be simplified by neglecting the inertial term, reducing the overall complexity of the system to Equations 5 and 6.

$$\hat{y}:\;mg-\frac{k_{e}q^{2}}{{4y}^{2}}-\frac{\dot{y}}{B}=0$$ (5)

$$\hat{x}:\;\dot{x}=u_{f}\left(y\right)$$ (6)

Whereas the horizontal particle trajectory follows a straightforward path, showcasing how the particle velocity aligns with that of the surrounding flow (Equation 6), the non-linear equation in the vertical direction (Equation 5) remains more complex and requires further analysis.

**FIGURE 3:**
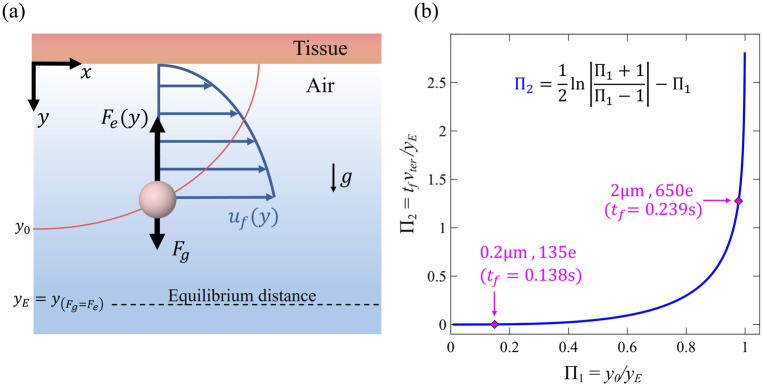
**(a)** Diagram illustrating an airborne particle near the wall of the model domain, released from initial distance *y*_*0*_. The particle is under the influence of electrostatic image charge forces $F_{e}\left(y\right)$ , gravitational sedimentation $F_{g}$ , and viscous drag resulting from the streamwise airflow velocity profile (i.e., laminar Poiseuille flow at low Reynolds number). **(b)** A theoretical non-dimensional plot of Π_2_ *versus* Π_1_, i.e., the two key dimensionless groups governing the final deposition time *t*_*f*_ under electrostatic attraction (see text for further details). The magenta diamond markers highlight two distinct charged particle cases analyzed in this study. The lower point corresponds to a small particle (0.2 μm, 135 e charge), for which gravitational effects are negligible, resulting in a falling time of approximately 0.138 s. The upper point represents a larger particle (2 μm, 650 e charge) with stronger gravitational forces, resulting in a falling time of approximately 0.239 s. Both particles are released from an initial height of *y*_*0*_ *= 0.025D*_*0*_.

We define the characteristic length scale $y_{E}$ as the “Equilibrium Distance” from the wall, where the electrostatic force balances gravity, i.e., $F_{e}\left(y_{E}\right)\equiv F_{g}$ (see Figure 3A). It is important to note that this length scale is specific to each particle’s size and charge levels and is given by $y_{E}=\sqrt{k_{e}q^{2}/4mg}$ . To better characterize electrostatic deposition, we define the normalized distance from the tissue as: $\eta =y\left(t\right)/y_{E}$ where the initial condition is given by $\eta_{0}=y_{0}/y_{E}.$ Hence, electrostatic deposition against gravity is expected only within the range 0≤η<1 . A careful simplification of Equation 6 (detailed in the Supplementary Material, in section “Analytical and Mathematical Derivation”) leads to the following form (Equation 7):

$$\hat{y}:\;\eta =\eta_{0}+\frac{v_{ter}t}{y_{E}}-\frac{1}{2}\ln\left(\frac{\eta -1}{\eta +1}\cdot \frac{\eta_{0}+1}{\eta_{0}-1}\right)$$ (7)

This non-dimensional equation is no longer a differential equation, making it significantly easier to solve and hence analyze particle behavior over time. We can write it dimensionally as:

$$\hat{y}:\;y_{\left(t\right)}=\overset{\text{free fall}}{\overbrace{y_{0}+v_{ter}t}}-\overset{\text{Electrostatic }}{\overbrace{\frac{y_{E}}{2}\ln\left(\frac{y_{\left(t\right)}-y_{E}}{y_{\left(t\right)}+y_{E}}\cdot \frac{y_{0}+y_{E}}{y_{0}-y_{E}}\right)\;}}$$ (8)

This Equation 8 describes displacement through two distinct terms. The first term on the left represents the classic free-fall sedimentation over time, while the nonlinear logarithmic term captures the opposing electrostatic attraction counteracting gravitational pull. Despite this clear separation, the Equation 8 is implicit in $y_{\left(t\right)}$ meaning an explicit expression for the particle’s position as a function of time is not straightforward. Yet, for very small particles, highly charged ones, or in a near proxinity to the tissue, gravitational settling is negligible (H≪Inc), the equilibrium distance $y_{E}$ extends toward infinity in comperison to $y\left(t\right)$ . Under these conditions $\left(\eta ,\eta_{0}\ll 1\right)$ , we can expand the logarithmic term in a Taylor series around zero up to the fourth order ( $O{\left(y\right)}^{5}$ ), leading to a simplified explicit results presented in Equation 9:

$$y\left(t\right)={\left(y_{0}^{3}-\frac{3}{4}kq^{2}Bt\right)}^{\frac{1}{3}}$$ (9)

This result offers a simplified yet direct analytical expression for the vertical displacement of the particle trajectory, providing a fundamental framework to understand how electrostatic charge influences deposition dynamics under Stokes drag.

An alternative approach to Equation (8) can be formulated using a non-dimensional framework. By considering the deposition condition $y\left(t_{f}\right)=0,$ the equation reduces to involve only two non-dimensional parameters: $\Pi_{1}=v_{ter}t_{f}/y_{E}$ , representing the free-fall distance normalized by the equilibrium distance, and $\Pi_{2}=y_{0}/y_{E}$ (analogous to $\eta_{0}$ ). This reformulation leads to Equation 10

$$\Pi_{1}=\frac{1}{2}\ln\left|\frac{\Pi_{2}+1}{\Pi_{2}-1}\right|-\Pi_{2}$$ (10)

Equation 10 provides a generalized expression for the final deposition time $t_{f}$ , accommodating any combination of charge, particle size and shape across all normalized initial conditions (Figure 3B). Upon reorganizing, the final deposition time is explicitly given in Equation 11:

$$t_{f}=\frac{y_{E}}{v_{ter}}\left(\frac{1}{2}\ln\left|\frac{y_{0}+y_{E}}{y_{0}-y_{E}}\right|-y_{0}\right)$$ (11)

### Statistical analysis

We conducted independent experiments for the combination of each particle size and group (i.e., charged vs neutralized). A three-way analysis of variance (ANOVA) was used to examine the effect of the experiment settings. The independent variables were the aerosol size (i.e., d_p_ ∈ {0.2, 0.5, 1.1} µm), the electrostatic charge group (i.e., q ∈ {Neutralized, Charged}), generation (i.e., Gen ∈ {G1,G2,G3}) and their interactions and state (constricted or Normal). The dependent variable was the Deposition Fraction (DF). In this work, we report both the p-values and the estimated effect sizes (β), where β represents the magnitude of change in DF and is expressed as a percentage to align with the units of DF itself. Additional details on the statistical analysis outcomes, beyond those presented in the Results and Discussion section, can be found in the Supplementary Material.

## Results and discussion

**FIGURE 4:**
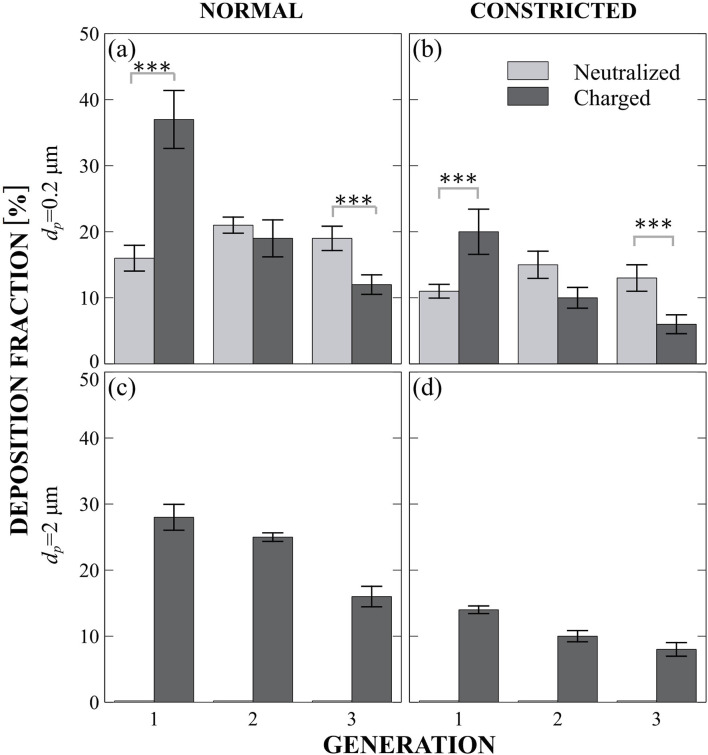
Four different cases of particles Deposition Fractions (DF) across airway generations in the *in vitro* model (see Figure 1). Rows distinguish between particle sizes (0.2 µm and 2 µm), and columns represent airway conditions (normal vs constricted). Light gray bars denote neutralized particle exposures, while dark gray bars indicate charged particle exposures. In each panel the *x*-axis indicates the airway generation, while the *y*-axis represents the corresponding DF. **(a)** Normal airway, *d_p_* =0.2 µm. **(b)** Constricted airway, *d_p_* =0.2 µm. **(c)** Normal airway, *d_p_* =2 µm. **(d)** Constricted airway, *d_p_* =2 µm.

In what follows, we present deposition data obtained from quantitative microscopy and numerical simulations, focusing on particle deposition in the microchannels. We extract from microscopy imaging the deposition fractions (DF) for each airway after completion of each exposure assay. A summary of the results is shown in Figure 4, where we present depositions according to particle sizes (Figure 4, rows) and regions: i.e., Constricted and Normal (Figure 4, columns). The histograms are normalized according to the total number of deposited particles. We discriminate between experiments where aerosols were neutralized (i.e., light gray histograms) and when we allow them bypass the neutralizer and maintain highly charged conditions (i.e., dark gray histograms). We note that the effect of lung generation (Gen. 1, 2 and 3) on deposition is not significant (p = 0.095), suggesting that it is not enough to conclude airway branching contributes to deposition in this particular model. This result is reasonable, as the airway generations examined in this study share similar characteristic length scales and flow velocities. However, in a larger model spanning additional airway generations, we would likely expect lung generation to exert a stronger influence on deposition patterns.

To ensure that the model reflects physiological flow, we conducted CFD simulations where we observe Hagen–Poiseuille-like flow across all the generations. In Figure 2a, we notice a minor overestimation of flow reduction due to constriction in the theoretical analysis (circles) compared to the CFD results (squares). This discrepancy likely arises from the minor pressure drop added by changes in airway diameter, as well as the bifurcation angles, that are omitted in the theoretical model. Moreover, in our analysis, each airway is modeled as an ideal rectangular resistor (see Supplementary Material Supplementary Figure S3a), based on the dimensions shown in Figure 2b. However, further down the airway, both the CFD results and the theoretical analysis converge as anticipated.

We recall that in our experiments, we examine a mild constriction (30%) with a moderate reduction in flow, which approximates the airflow reduction in the constricted regions about two-thirds of that in an open healthy airway and hence less deposition expected there. The influence of lung state (Normal vs Constricted) on deposition is found to be highly significant (p < 0.0001, β = 8%), underscoring that airway narrowing indeed significantly alters deposition patterns. This finding aligns with clinical studies showing that constricted airways are associated with impaired particle transport and reduced deposition efficiency (Diaz et al., 2023; Xu et al., 2022). It is important to highlight how the characteristic length scale differs in the constricted region *versus* normal in the model (Figure 3d); a point we will revisit due to the greater sensitivity of electrostatic forces to length scale compared to other mechanisms. Particle size has a significant impact on deposition in the model (p < 0.0001, β = 14% for 0.2 μm–2 μm), confirming that different particle sizes lead to distinct DF. This finding is consistent with existing literature across the aerosol drug delivery community (Ijsebaert et al., 2001; Darquenne, 2012; ICRP, 1994).

### Role of electrostatics *versus* diffusion

Upon examining the smallest particle size (0.2 μm), deposition primarily occurs due to Brownian motion (Figures 4a,b) since Pe^−1^ is eight times larger than H. When neutralized (light gray), deposition in the normal region remains constant, plateauing at ∼20% across the three generations. As expected, in the constricted region, there is less flow with available particles to deposit, leading to a lower deposition rate of around ∼12% per generation. However, when the particles are charged, a different deposition pattern emerges. Charge has a strong and highly significant effect on deposition fraction DF (p < 0.0001, β = 15%), indicating that electrostatic forces play a major role in particle behavior in our model. We observe a screening effect, where deposition is dominated in the first generation, and this effect extends to the rest of the model. Screening refers to particles that are unlikely to reach more distal regions because they first collide with nearby surfaces, effectively “screening” the regions farther downstream. This results in the streamwise depletion of particles. The phenomenon of “diffusional screening” is well known in the respiratory system (Felici et al., 2003; Hofemeier et al., 2016). Here, we clearly observe that the superposition of Brownian motion and electrostatic forces leads to screening in proximal airways. The interaction between charge and generation is highly significant (β ∼ −18% for Gen 2 and β ∼ −22% for Gen 3 with respect to Gen 1,p < 0.0001 for both), demonstrating how charge-driven screening influences DF. This effect has also been observed in a recent *in vitro* electrostatic study in the acinar region (Bessler et al., 2023). When comparing the charged cases between normal and constricted regions, the DF ratios remain similar, reflecting the mild degree of constriction applied in this particulate study. As a result, no major change in deposition patterns was observed. However, with more severe constriction, we would indeed expect enhanced electrostatic effects, as the reduced characteristic length (*L*_*c*_) favor the electrostatic attraction (Inc⋅Pe $\propto L_{c}^{-1}$ ; Bessler and Sznitman, 2024).

**FIGURE 5:**
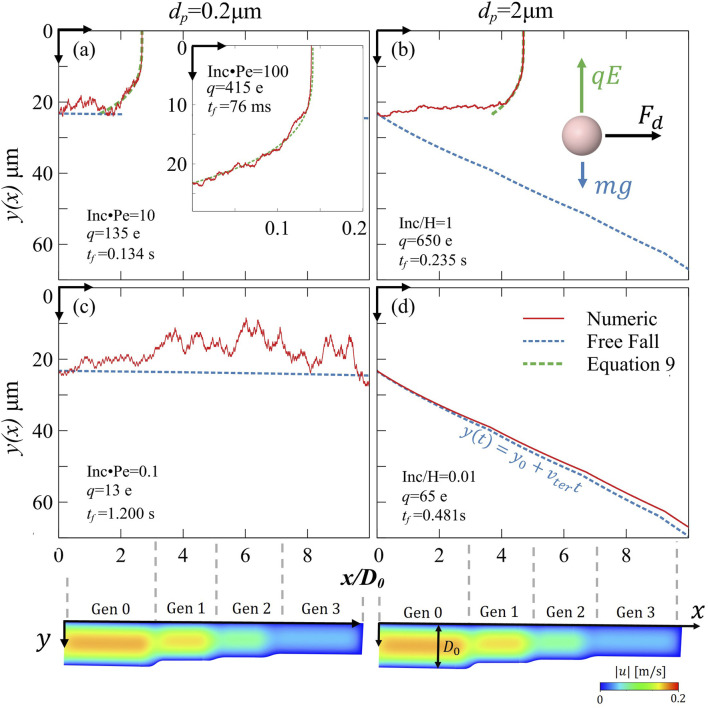
Numerical simulation of particle trajectories within the XY plane of the model. The **red** line represents the actual particle trajectory from the simulation (i.e., including electrostatics sedimentation and diffusion), while the dashed lines give a theoretical comparison: **blue** indicates the free-fall trajectory, **green** indicates electrostatics against drag (Equation 9). The vertical axis *y(x)* shows the particle’s distance from the top wall, while the horizontal axis *x/D*_*0*_ represents the normalized upstream direction across four generations. Below the horizontal axis, CFD velocity magnitudes for visual guide reference (see Supplementary Material Supplementary Figure S3b for CFD 3D view). The rows distinguish between cases where electrostatic forces dominate (first row) and cases where charge levels are insignificant (second row). In the lower left corner of each simulation, the chosen specific charge (*q*) and the resulting final time (*t*_*f*_) (indicating the time until deposition or model exiting) are displayed. The figure spans five orders of magnitude in the non-dimensional parameter for induced charge (**Inc) compared to other mechanisms**, illustrating the transition in dominant forces across different regimes: **(a)** Electrostatic forces are non-negligible for the particle motion **Inc·Pe = 10**, where subfigure shows even **more** dominating electrostatics: **Inc·Pe = 100**. **(b) Inc/H = 1** implying that electrostatic and gravitational forces are almost balanced (subfigure illustrates the forces acting on the particle). **(c) Inc·Pe = 0.1** showcasing a diffusion-dominated regime. **(d) Inc/H = 0.01** showcasing a sedimentation-dominated regime.

To gain further insight, we analyzed particle trajectories using numerical simulations. Figure 5 presents five representative cases that are anticipated to occur in our model, considering the given particle sizes and expected charges (Forsyth et al., 1998; Whitby and Liu, 1968). Each particle in our numerical simulations experiences the same four main forces (Figure 2a; Supplementary Figure S5b inset): an electrostatic force directed upward toward the top of the model ( $-\hat{y}$ ), gravitational force acting downward ( $\left.+\hat{y}\right)$ , Brownian random diffusion, and viscous drag, which mitigate it vertical motion and primarily pulls the aerosol downstream in the $\hat{x}$ horizontal direction in a convective manner. It is important to recognize that the characteristic velocity in the bronchioles *U*_*c*_, which represents the convective nature of airflow, appears in the denominator of each dimensionless term (Inc, H, Pe^−1^∝ *U*_*c*_^*−1*^). Since *U*_*c*_ is significantly larger than the remaining parameters, these terms become much smaller in magnitude (Inc, H, Pe^−1^≪1), supporting the phenomenon of poor aerosol deposition in the bronchioles due to the dominance of convection over other mechanisms (ICRP, 1994). Hence, to better understand the interplay between electrostatic forces (Inc) and other deposition mechanisms (H, Pe^−1^), we compare the relative contributions of electrostatic effects to the dominant transport mechanisms. For the largest particles (2 μm), where Pe^−1^≪H, the ratio Inc/H compares electrostatic attraction *versus* gravitational settling (Figures 5b,d). Conversely, for the smallest particles (0.2 μm), where H ≪ Pe^−1^, the term Inc⋅Pe contrasts electrostatic with random diffusive motion (Figures 5a,c). These ratios effectively eliminate the dependence on *U*_*c*_, allowing us to isolate mechanism interplay from overwhelming convection effect. This approach provides a clearer framework for analyzing the physical mechanisms governing aerosol deposition and enhances our discussion of electrostatic effects in the deep lung.

Figure 5a highlights the dominance of electrostatic forces over diffusion (Inc⋅Pe ∼ 10 and inset Inc⋅Pe ∼ 100), whereas Figure 5c provides a comparison by illustrating deposition mechanisms when electrostatic effects are negligible in comparison to diffusion (Inc⋅Pe = 0.1). Note that all particles were released from a height *y*_*0*_, corresponding to 5% of the radius of generation 0, coinciding with the characteristic length scale (*L*_*c*_ ∼ *y*_*0*_). This choice satisfies the image charge assumption (*y*_*0*_ << *D*_*0*_) and follows the conventional approach for examining electrostatic effects through dimensionless analysis (Finlay, 2001). This distance *y*_*0*_ presents ∼10% of the total airway volume (or cross-section) populated by aerosols prone to deposition. We immediately observe a clear difference between the two cases for 0.2 µm particles. When electrostatic forces are superimposed with Brownian motion (Figure 5a), the particle trajectory is not entirely random; rather, it oscillates slightly around a dominant, sharply directed path toward the tissue, dictated by electrostatic attraction (dashed green line). This sharp path can be predicted using our analytic solution (Equation 9) which closely matches numerical simulations, with even greater agreement observed in the inset (Inc⋅Pe ∼ 100). This equation closely approximated the trajectories of small-mass particles (e.g., 0.2 μm ) where gravity is almost neglected (H < Pe^−1^) or for highly charged particles in close proximity to the tissue, where other forces become insignificant.

The charged particles exhibit very short airborne times, with deposition occurring within tenths of a second (e.g., 0.134 s). In this regime (Inc·Pe ∼ 10), diffusion can either hinder or enhance deposition time when combined with electrostatic effects (Figure 5a). Our non-dimensional plot (Figure 2b), which also accounts for the minor influence of gravity, predicts a deposition time of *t*_*f*_ = 0.138 s, closely matching with the numerical simulations. As implied by Equation 9, the higher charge presented in Figure 5a inset, results in even shorter deposition times, reaching approximately 75 ms. In contrast, neutralized particles (Figure 5c) remained airborne for over a second and ultimately escaped the model, despite reaching 10 µm away from the tissue due to Brownian motion. A slightly higher charge would likely have ensured deposition and close this gap. These results highlight how electrostatics effects accelerate screening behavior and enhance deposition efficiency in the proximal generations of the bronchioles.

Equation (9) further highlights the critical role of mechanical mobility *B*, derived from the particle’s size and shape. Mechanical mobility playing a role as significant as the charge squared itself. This finding aligns well with clinical *in vivo* observations in humans, where DF for similar sizes (i.e. 0.3, 0.6 and 1 µm) has been found to follow a similar scaling relationship, $DF\propto {\left(q^{2}B\right)}^{\frac{1}{3}}$ (Melandri et al., 1983). This analytical result concurs with our finding of highly significant interaction between particle size and charge (p < 0.0001, for 0.2 μm β∼15% for Gen 1, see Supplementary Material Supplementary Table S6 for more) since *B* is linear with the particle diameter *d*_*p*_. In the future, more complex CFD models that include real lung anatomies (Koullapis et al., 2016) can leverage our approach to predict deposition location in the lungs based on the airborne time *t* of these two particle dynamic approaches.

We acknowledge that our airway-on-chip platform is based on a planar geometry, which does not fully capture the complex three-dimensional architecture of the human lung. However, the two primary mechanisms examined in this study, electrostatic attraction and diffusion, are largely independent of spatial orientation. Electrostatic forces follow electric field lines toward the nearest surface, while diffusion is isotropic and random. Therefore, in clinically relevant scenarios involving small, charged particles under low to moderate flow conditions, the deposition patterns observed in our planar model are anticipated to approximate sufficiently closely those in a realistic 3D airway structure. Nevertheless, we recognize that in regions with higher airflow velocities (e.g., the proximal bronchi), inertial effects may become more pronounced and sensitive to airway geometry. Additionally, in the distal bronchioles, gravitational settling becomes increasingly important for heavier particles, and the 3D orientation and curvature of the airways are likely to influence deposition. This is addressed below.

#### Role of electrostatic *versus* sedimentation

We now turn our attention to the largest particles, i.e. 2 μm. Recalling that H·Pe ∼ 235, these particles, when neutralized, are governed almost entirely by gravitational pull. In this regime, the planar orientation of our model becomes particularly relevant, as gravity acts in a fixed direction, and the absence of 3D airway curvature may influence the observed deposition patterns for clinical interpretation of the results. In order to distinguish the electrostatic forces from gravitational effects in our experiments, we conducted experiments with the model turned upside-down such that the ITO-coated surface is at the top, hence electrostatic may be interpreted as a “lifting force” against the downward pull of gravity. As expected, in the neutralized case, no deposition occurred on the top even when we extended the exposure time (see missing bars in Figures 4c,d). To validate this, as discussed in the Supplementary Material, we repeated the experiment with the model flipped to align with gravity and observed deposition biased by gravity direction, both with and without charge yields significant deposition which is hard to distinguish (see Supplementary Material Supplementary Figure S4c and S4d). This confirms the significant role of gravity in the bronchiolar region, as suggested previously (Ma and Darquenne, 2012). As expected, gravitational deposition occurs only in the direction of the gravitational pull. This can be visually shown in our numerical analysis of particle trajectories (see Figure 5d, Inc/H ∼ 0.01) where particles do not reach deposition at the top of the model when electrostatic forces and diffusion are absent. The particle follows its free-fall trajectory (see Figure 5d, dashed blue line) away from the tissue, with only slight deviations caused by weak diffusion. Its airborne time is approximately half a second (0.48 s), which is shorter compared to the 0.2 µm diffusive particle (1.200 s). This difference arises because the free-falling particle enters closer to the channel centerline, leading it to gain horizontal velocity as it moves into the high-speed region of the parabolic Poiseuille flow profile, resulting in a faster outward convection.

When these same 2 μm particles are charged, they have a chance to deposit. A distinct electrostatic-driven screening effect emerges leading to deposition within the bronchial model (Figure 4b). This suggests that electrostatic forces are enough to counteract gravity, encouraging greater bronchiolar deposition, even in regions where gravitational settling is unlikely to occur. We recall that the characteristic length scale $y_{E}$ represents the “Equilibrium Distance” from the wall at which the electrostatic force balances gravity. This length scale is crucial for understanding particle dynamics under the influence of both electrostatic forces and gravitational effects. Mathematically, it serves as a so-called separatrix, distinguishing between two distinct scenarios: (i) deposition due to electrostatic attraction *versus* (ii) convective transport through the channel to the next-generation. Most likely, in our case, this results in $y_{E}\;∼25\;\mu m$ (Forsyth et al., 1998; Whitby and Liu, 1968). Based on our *in-vitro* deposition findings (Figures 4c,d), we can infer that regions where $y\left(t\right)<y_{E}$ are populated with aerosols that are most likely deposited due to electrostatic contributions. In comparison to the screening observed for the 0.2 µm particles, the gradient of the screening is less intense here. This can be attributed to the fact that while Inc⋅Pe is not dependent on particle size, the ratio Inc/H ∝ *d*_*p*_^−3^ is highly size-dependent. For the larger 2 µm particles, this strong dependence reduces the effectiveness of electrostatic deposition, as their increased dimensions hinders the influence of electrostatic forces. This is clearly shown and aligns with an *in vitro* study (Bessler et al., 2023) and an additional upper airway CFD study, where Inc/Stk ∝ *d*_*p*_^−3^ follows the same dependency (Koullapis et al., 2016). As a result, more substantial deposition in proximal airways occurs for smaller particles, reinforcing the weight of electrostatic forces for smaller aerosols (Koullapis et al., 2016). Accordingly, a highly significant interaction between particle size and charge (p < 0.0001, for 2 μm β∼21% for Gen 1, see Supplementary Material Supplementary Table S6 for more), indicating that the effect of particle size on deposition depends on charge, and *vice versa*. Hence, charge does not influence all particle sizes uniformly, certain sizes are more affected by electrostatic forces than others.

Although electrostatics generally increased overall targeted bronchiolar deposition, it is important to note that across all tested combinations of charge states (neutralized and charged) and particle sizes (0.2, 0.5, and 2 µm), no significant increase in deposition was observed within the constricted regions compared to the normal airways. This finding indicates that, within the limited range of particle sizes and electrostatic charge levels explored in this study, we did not identify conditions that preferentially enhance deposition in constricted regions. Specifically, for 2 µm particles, deposition in the constricted airway was consistently about half of that observed in the normal airway, regardless of charge. These results underscore the ongoing challenge of achieving efficient, targeted aerosol delivery to the bronchioles, especially in distinguishing deposition behavior between constricted and non-constricted geometries. Future studies should explore a broader range of particle sizes, electrostatic charge levels, and airway constriction severities, as this work scope focused mainly on the effect of electrostatics in the bronchiole region and consider only one case of a mild constriction. Given that electrostatic forces become increasingly dominant at smaller length scales, we hypothesize that a promising window of opportunity remains to be uncovered—one that could enable selective targeting of diseased airways through optimized charge and size combinations.

In Figure 5b, we examine the particle trajectory under conditions where electrostatic and gravitational forces are of comparable magnitude (Inc ∼ H), as suggested by Equation (8). The simulation reveals that charged particles can ascend against gravity and deposit on airway surfaces that would otherwise remain unaffected. This behavior aligns with our *in vitro* findings (Figure 4a), where charged particles exhibited enhanced deposition compared to their neutralized counterparts. Notably, since the release height $y_{0}$ is close to the equilibrium distance ( $y_{E}\;∼25\%\;of\;D_{0}$ ), the particle spends most of its airborne time hovering near this equilibrium position. However, beyond a critical threshold, the electrostatic force becomes strong enough to overcome gravitational pull entirely, and the particle trajectory converges with the analytical prediction of Equation (9) (green dashed line). As a result, the final velocity of the particle moving upward against gravity surpasses its free-fall terminal velocity by three orders of magnitude ( $v_{ter}\ll v_{f}$ ). In summary, with clinical relevance, electrostatic forces expand the effective bronchial tissue area available for aerosol delivery by enabling particles to reach the top surfaces throughout the airway cross-section, and not only those who favor gravity direction. Hence, electrostatics demonstrate substantial potential for enhancing drug delivery efficiency, particularly in regions where conventional mechanisms fall short.

As a final step, we demonstrated the non-dimensional dependency (Figure 2b) that applies to all deposition scenarios involving electrostatic forces *versus* gravity. The result of our theoretical non-dimensional plot perfectly aligns with the deterministic trajectory and the one predicted by the numerical simulation, yielding a deposition time of $t_{f}$ = 0.239 s in both approaches. This framework serves as a valuable and comprehensive summary, capturing the interplay between particle shape, size, charge, and initial conditions through two dimensionless parameters: *y*_0_/*y*_*E*_ and *v*_ter_ *t*_f_/*y*_*E*_. This relationship provides an effective tool for estimating the deposition time *t*_*f*_, which can be further related to the convective timescale. This representation also accurately predicts deposition times for our specific cases, such as 0.2 and 2 µm particles, showing strong agreement with our numerical solutions. Notably, deposition time approaches infinity when particles are uncharged or if they are initially positioned near the equilibrium stagnation point $y_{E}$ . The initial height $y_{0}$ plays a crucial role in determining deposition time. In constricted airways, particles are more likely to start from a lower initial height, smaller distance leading to shorter deposition periods. Additionally, as observed, the particle’s terminal velocity $v_{ter}$ , influenced by its shape, remains a key factor in deposition dynamics. While our simulations assume spherical particles, it is important to note that alternative particle shapes could exhibit significantly different behaviors (Kleinstreuer et al., 2008; Kleinstreuer and Zhang, 2010). For example, long, straight fibers interact differently with the drug medium (Asgharian and Yu, 1988; Harris and Timbrell, 1975; Shachar-Berman et al., 2018). Asbestos fibers, for instance, with lengths of 50–200 μm, are known to deposit extensively in the lungs and cause severe health conditions (Timbrell, 1965; Donaldson et al., 1989). In the context of electrostatic, *in vivo* studies in rodents have found much higher acinar deposition in charged asbestos fibers compared to neutralized ones (Davis et al., 1988). This suggests that combining non-spherical shaped particles with electrostatic calls for future investigation (Bessler and Sznitman, 2024). While most accepted approaches neglect the role of electrostatic effects, a growing body of evidence suggests that they play a significant role in altering deposition (Kwok and Chan, 2009).

## Conclusion

Using a novel *in vitro* airway-on-chip model, we systematically explored how electrostatic charge alters deposition patterns compared to classical mechanisms such as Brownian motion and gravitational sedimentation. Our results support that charge plays a dominant role in aerosol deposition at the bronchiole length scale, particularly for smaller aerosols, where electrostatic attraction accelerates deposition and causes diffusion screening to occur earlier. For neutralized larger particles, gravity primarily dictates a downward trajectory, but when charged, electrostatic forces influence their motion by defying gravity and promoting deposition in wider areas such as the “ceilings” of the airway wall. Hence, an increase in charge enhances the effective deposition region in bronchioles for 2 µm particles, while for smaller particles, it accelerates their deposition by electrostatic screening in this region. Additionally, our numerical simulations and analytical derivations offer a valuable step for overcoming the complexity of introducing electrostatics into particle dynamics. They provide a visual representation of the transport mechanisms and highlight key findings, such how the aerosols’ mechanical mobility can counteract electrostatic attraction, potentially allowing aerosols to penetrate deeper into the distal lung regions. These findings have relevant implications for inhaled drug delivery, suggesting that electrostatic manipulation along with size could serve as a tunable parameters for optimizing deposition in targeted airway regions, particularly where traditional aerodynamic strategies fail.

This suggests the need for further investigation into a broader variety of constriction severity and charge-diameter combinations that may selectively favor deposition in diseased airways, offering a potential avenue that may improve therapeutic targeting in obstructive lung diseases beyond the limited cases examined in this study (Bessler and Sznitman, 2024). An open question remains regarding the spatial distribution and the accumulative effect of electrostatics throughout the lungs. Some studies suggest that electrostatic forces may enhance deposition in the upper airways, raising concerns that charged particles may be lost before reaching critical therapeutic regions like the bronchioles and alveoli (Melandri et al., 1983; Koullapis et al., 2016; Azhdarzadeh et al., 2014). Alternatively, the finding here may suggest that the bronchioles themselves may act as a screening mechanism, limiting deposition in the deeper acinar regions. To fully address this question, multiscale models are needed to identify the window of opportunity in which charged aerosols can provide therapeutic benefit. Although the effect of electrical charge has been recognized for nearly a century (Wilson, 1947), the role of electrostatic forces in pulmonary deposition remains fundamentally underexplored. Overshadowed by more widely studied mechanisms such as impaction, sedimentation, and diffusion, electrostatics has often received limited attention, potentially hindering its broader application in aerosol drug delivery. To fully harness its potential, a deeper understanding is needed of the conditions under which electrostatic forces can meaningfully influence particle behavior throughout the respiratory tract. Addressing these overarching questions may require the development of multiscale lung models capable of capturing the cumulative effects of electrostatic interactions across multiple airway generations (Kassinos and Sznitman, 2024). Future studies should focus on expanding the range of charge distributions, incorporating asymmetric airway geometries, and exploring alternative particle morphologies, including fibers and aggregates, to better capture real-world deposition dynamics. Ultimately, this work reinforces the importance of electrostatics as a fundamental mechanism in pulmonary aerosol transport.

## Data Availability

The original contributions presented in the study are included in the article/Supplementary Material, further inquiries can be directed to the corresponding authors.
