# Supplementary material for: Small scale electrostatically-driven aerosol deposition in airway-on-chip models of bronchial constriction
Source: Front Physiol. 2025 Sep 16;16:1621177. doi: 10.3389/fphys.2025.1621177 (PMC12482922; doi:10.3389/fphys.2025.1621177)
Supplement: Supplementary file 1 [file DataSheet1.docx]

Small scale electrostatically-driven aerosol deposition in *airway-on-chip* models of bronchial constriction

Ron Bessler^1^, Tirosh Mekler^1^, Rami Fishler^1^, Oshri Farhana^1^, Sigal Dhatavkar^1^, Tamar Daniel^1^, Bar Kalifa^1^, Kenichiro Koshiyama^2^, Netanel Korin^1^ & Josué Sznitman^1^

^1^ Department of Biomedical Engineering, Technion – Israel Institute of Technology, Haifa, Israel

^2^ Graduate School of Technology, Industrial and Social Sciences,  Tokushima University, Tokushima, Japan

# Supplementary Material

## Experiment & Model design and fabrication

Figure S1 presents the experimental setup inside the chemical hood, along with some of the crucial apparatus components involved in the model design.


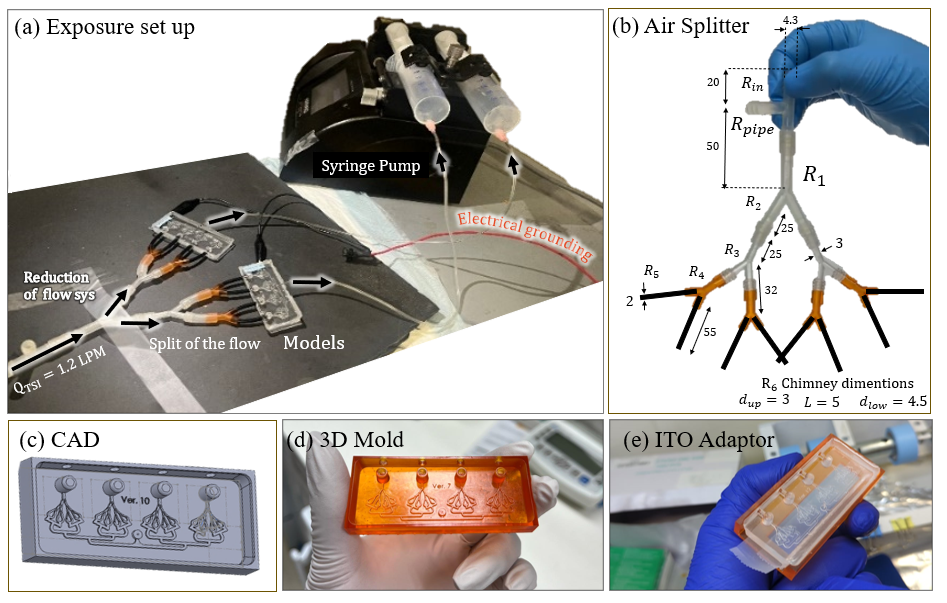


Figure S1 (a) Aerosol exposure setup based on an aerosol generator that feeds the model. (b) To reduce the high velocity of the airflow, we designed an air splitter that diverts approximately 73% of the flow and then evenly distributes the remaining air between the microchannels (dimensions in mm). (c) CAD of our mold which includes four bronchioles microchannel models, each with a 30% constriction in one branch (compared to the normal branch). From the aerosol generator, the airflow is reduced to 40 ml/min, which feeds the inlet. Using a syringe pump, ~6% of the flow (2.5 ml/min) is directed into the microchannel at generation 0 (G0), while the remaining air is expelled through the vertical bleeding chimney. The low Re air entering the microchannel at G0 splits again at G1, which consists of two different hydraulic diameters: a normal-sized branch receiving ~80% of the flow and a constricted branch receiving only ~20%. (d) The 3D mold used for model fabrication. (e) Adapter to ensure the model sits correctly on top of the ITO layer during the stamping process.

### Conductive Layering of the Microchannel

We applied a conductive indium tin oxide (ITO) coating to our microscope slide, following previous methodologies [1]. The ITO pattern was selectively deposited only on regions corresponding to the airway microchannels (see Fig. S2).


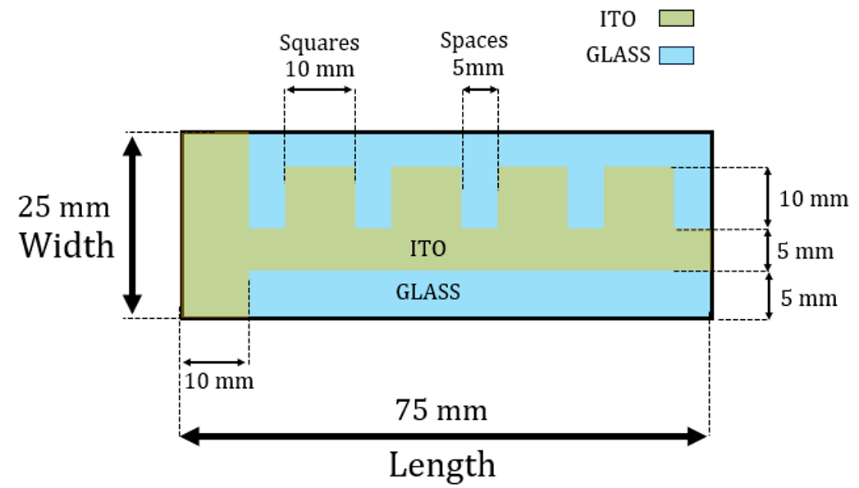


Figure S2: The bottom part of the model is a microscope slide (blue) covered by a specific pattern of ITO (gold) to ensure electrostatic-induced charge effects on the lower surface. The entire model is coated with the same ITO layer, and the left side provides a convenient electrical grounding zone.

A more comprehensive discussion of the electrical time scale analysis, which lays in the ability of tissue charges to adapt their orientation in response to an external electric field such as one generated by a charged airborne particle in the lumen, is a consequence of solving the current continuity equation which supplies bellow:

|  | $\vec{\nabla}\cdot\vec{j}+\frac{\partial\rho_{e}}{\partial t}=0$ | (1) |
| --- | --- | --- |

Where $\vec{J} \left( \frac{e}{{s\cdot m}^{2}} \right)$ is the current density and $\rho_{e}\left( \frac{e}{m^{3}} \right)$ is the volume charge density. Using Gauss law, and Ohms law where $\sigma$ is the tissue conductivity, we can simplify the divergence of the current density $\vec{\nabla}\cdot\vec{j}$ .

Gauss law:

|  | $\vec{\nabla}\cdot\vec{E}=\frac{\rho_{e}}{\varepsilon}$ | (2) |
| --- | --- | --- |

Using Ohm's law:

|  | $\sigma\vec{E}=\vec{J}$ | (3) |
| --- | --- | --- |

We can substitute the electric field:

$$\vec{\nabla}\cdot\left( \frac{1}{\sigma} \vec{J} \right)=\frac{\rho_{e}}{\varepsilon} \to\vec{\nabla}\cdot\vec{J}=\frac{\sigma}{\varepsilon}\rho_{e}$$

Substitute back into current continuity equation, we solve an unsteady ODE for $\rho_{e}(t)$:

|  | $\frac{\sigma}{\varepsilon}\rho_{e}+\frac{\partial\rho_{e}}{\partial t}=0$ | (4) |
| --- | --- | --- |

Its solution is:

|  | $\rho_{e}(t)=\rho_{0}e^{-t/\tau_{e}}$ | (5) |
| --- | --- | --- |

where $\rho_{0}$ is a charge density constant and $\tau_{e}$is the electrical time constant defined as:

|  | $\tau_{e}=\frac{\varepsilon}{\sigma}$ | (6) |
| --- | --- | --- |

The manufacture supplies the sheet resistivity and not the conductivity of the glass. Sheet resistance is a measure of the resistance of a square piece of thin material with contacts made to two opposite sides. It is typically used to characterize the electrical properties of thin films that have uniform thickness, such as those created through semiconductor doping, metal deposition, resistive paste printing, and glass coating. Common applications include assessing doped semiconductor regions (like silicon or polysilicon) and the resistors used in thick-film hybrid microcircuits. The primary advantage of sheet resistance over traditional resistance or resistivity measurements is its consistency under scaling, allowing for direct comparisons between devices of varied sizes. Electrical resistance is defined as:

|  | $R=\frac{\rho l}{A}$ | (7) |
| --- | --- | --- |

Where$\rho$ is the material's resistivity we are aiming to find, $l$ is the length of the resistor, and $A$ is the cross-sectional area of the resistor, which can be divided into its width (w) and thickness (t):

|  | $R=\frac{\rho l}{wt}$ | (8) |
| --- | --- | --- |

By defining the sheet resistance as:

|  | $R_{s}=\frac{\rho}{t}$ | (‎09) |
| --- | --- | --- |

Hence resistance can now be expressed as:

|  | $R=R_{s}\frac{l}{w}$ | (10) |
| --- | --- | --- |

When the geometry is known, sheet resistance can be measured using a four-terminal sensing method (four-point probe measurement) or non-contact eddy-current-based testing devices. This measurement is crucial for evaluating and comparing the electrical properties of thin film materials in various technological applications. In our current study it was measured to be $R_{s}\sim10 \Omega/m^{2}$ while the microscope slide thickness is $t\sim1 \mathrm{mm}.$ Hence we could deduce the resistivity by:

|  | $\rho=R_{s}\cdot t=10\cdot{10}^{-3} \to\rho={10}^{-2} \Omega/m$ | (‎011) |
| --- | --- | --- |

For finding the conductivity:

|  | $\sigma=\rho^{-1}=100\frac{S}{m}$ | (12) |
| --- | --- | --- |

This value is much larger than the common microscope glass conductivity ($\sigma_{glass}\sim{10}^{-11} S/m)$ and thus is enough to account for image charge phenomena to take place. Units check: $\tau_{e}=\frac{\left[ \frac{F}{m} \right]}{\left[ \frac{1}{\Omega m} \right]}=\frac{\left[ F \right]}{\left[ 1/\Omega\right]}=\frac{\left[ F \right]}{\left[ F/s \right]}=[s]$.

In Table S1 we summarize the different electrical properties which include conductivity, dielectric constant, and the electrical time constant, where $\varepsilon_{0}$ is the dielectric constant of vacuum $8.85\times{10}^{-12} \left( \frac{F}{m} \right)$.

| Material | Conductivity  $\sigma\left[ S/m \right]$ | Dielectric Constant $\varepsilon[F/m]=\varepsilon_{r}\varepsilon_{0}$ | Electrical time constant  $\tau_{e}[s]=\frac{\varepsilon}{\sigma}$ |
| --- | --- | --- | --- |
| Glass | $\sigma_{\mathrm{Glass}} \sim{10}^{-11}$ | $\varepsilon_{\mathrm{Glass}} \sim5 \varepsilon_{0}$ | $44.25 [s] \sim1[min]$ |
| $H_{2}O$ | $\sigma_{H_{2}O} \sim1$ | $\varepsilon_{H_{2}O} \sim80 \varepsilon_{0}$ | $\sim7\times{10}^{-10}[s]$ |
| ITO | $\sigma_{\mathrm{ITO}}\sim{10}^{2}$ | $\varepsilon_{\mathrm{ITO}} \sim3 \varepsilon_{0}$ | $\sim{10}^{-13}[s]$ |

Table S1 Electrical time constants.

To ensure that the charge density $\rho_{e}(t)$ within the microchannel aligns with the tissue's behavior, it is essential to establish a similar relationship between the electrical time constant $\tau_{e}$ and other time constants associated with the flow, denoted as $\tau_{\mathrm{flow}}$. There are various alternative time scales that characterize flow, including the breathing rate ($\tau_{\mathrm{Breathing}}=4 [s]$), the advective time scale of the airway ($\tau_{\mathrm{advective}}=\frac{L_{c}}{U_{ave}}=\frac{50\mu m}{0.1 m/s}={5\times10}^{-4} [s]$), and even the particle relaxation time ($\tau_{p}=\rho_{p}d_{p}^{2}/18\mu_{f}\sim{10}^{-6} [s]$). However, regardless of the specific choice among these alternatives, it is important to note that electrostatic effects within the tissue occur on a much shorter timescale compared to the flow ($\tau_{e}\ll\tau_{flow}$). Therefore, any selection that adheres to this relationship will be suitable for faithfully replicating the anticipated electrostatic effects within the lung, as we aim to achieve in our research. Conductive materials, including metals, are viable choices for this purpose [2]. However, in our research, we selected ITO due to its transparency feature, which ensures minimal interference with our particle measurements.

### Airway Resistance Calculations

The resistance of each airway generation was calculated assuming fully developed Hagen–Poiseuille flow within a square cross-section, employing the expression:

| $R_{i}=28.41\frac{\mu_{air}L_{i}}{D_{i}^{4}}$ | (13) |
| --- | --- |

Where $L$ is the airway length,$D$ is the hydraulic diameter, and $\mu_{air}$ is the dynamic viscosity of air. The constricted region of the model was accounted for by applying a 30% reduction in diameter, as detailed in the table presented in Figure 3b of the main manuscript. A schematic diagram illustrating the resistor network construction used in the model is provided in Figure S3a. For comparison, the CAD model shown in Fig. S3b presents the velocity profile in the XY plane, based on the ANSYS fluid dynamics simulation featured in the main paper.


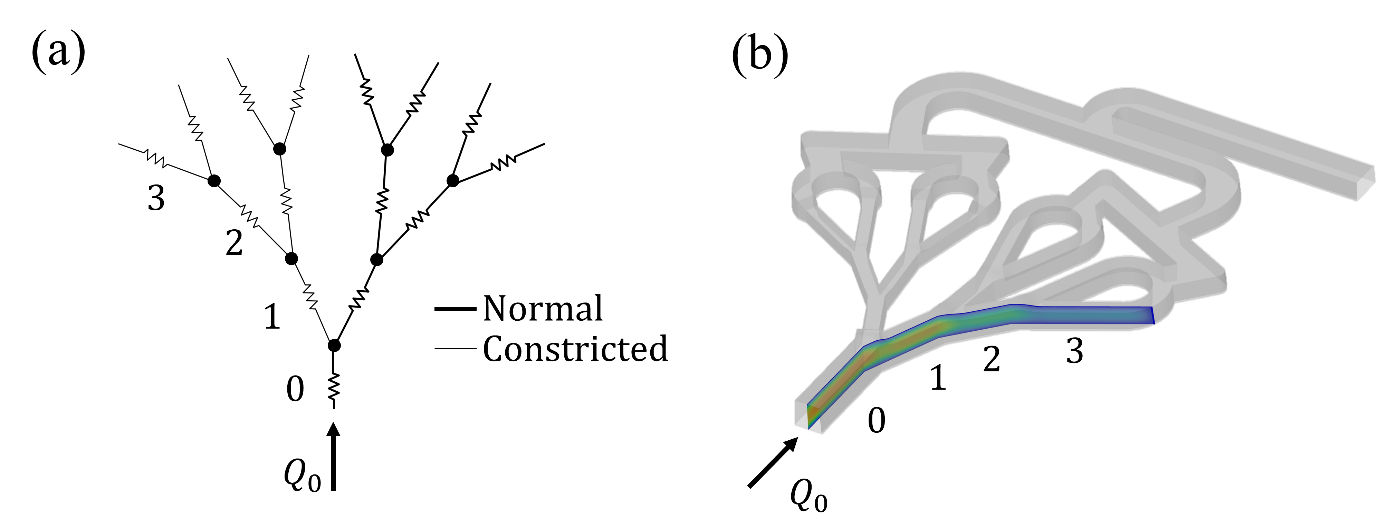


Figure S3 Microchannel flow analysis presented in two configurations: (a) A schematic diagram of an idealized electrical circuit where each airway generation is modeled as an ideal flow resistor. Normal-sized airways are represented by thick lines, while constricted segments are shown with narrower lines. (b) Comparison to the CAD model with a corresponding computational fluid dynamics (CFD) simulation of the XY plane, demonstrating the development of Hagen–Poiseuille flow within the microchannel network.

## Analytical & Mathematical Derivation

### Integral Solution

In the particle dynamics we reach the following term:

$$\frac{v_{ter}t}{y_{E}}=\int\frac{1}{1-\frac{1}{\eta^{2}}}d\eta$$

To derive an analytic theoretical expression for the particle’s trajectory, we solved the presented integral:

| $\int\frac{1}{1-\frac{1}{\eta^{2}}}d\eta= ?$ | (14) |
| --- | --- |

$$\int\frac{1}{1-\frac{1}{\eta^{2}}}d\eta=\int\frac{1}{1-\frac{1}{\eta^{2}}}\underset{=1}{\underbrace{\frac{\eta^{2}}{\eta^{2}}}}d\eta=\int\frac{\eta^{2}}{\eta^{2}-1}d\eta$$

By adding and subtracting one we can split the expression:

$$=\int\frac{\eta^{2}\overset{=0}{\overbrace{+1-1}}}{\eta^{2}-1}d\eta=\int\underset{=-1}{\underbrace{\frac{\eta^{2}-1}{\eta^{2}-1}}}+\frac{1}{\eta^{2}-1}d\eta=\int\frac{1}{\eta^{2}-1}+1 d\eta=\int\frac{1}{\eta^{2}-1}d\eta+\underset{=\eta}{\underbrace{\int1 d\eta}}$$

$$=\underset{=?}{\underbrace{\int\frac{1}{\eta^{2}-1}d\eta}}+\eta+C$$

Solving the first integral, we factor the denominator and perform partial fraction decomposition:

$$\int\frac{1}{\eta^{2}-1}d\eta=\int\frac{1}{\left( \eta-1 \right)\left( \eta+1 \right)}d\eta$$

$$=\int\frac{1}{2\left( \eta-1 \right)}-\frac{1}{2\left( \eta+1 \right)}d\eta$$

$$=\frac{1}{2}\left[ \int\frac{1}{\left( \eta-1 \right)}d\eta-\int\frac{1}{\left( \eta+1 \right)}d\eta\right]$$

we substitute $u=\eta\pm1 \to du=d\eta$ and get a standard integral:

$$\int\frac{1}{\eta\pm1}d\eta=\int\frac{1}{u}du=\ln\left( u \right)=ln(\eta\pm1)$$

$$=\frac{1}{2}\left[ \underset{ln(\eta-1)}{\underbrace{\int\frac{1}{\left( \eta-1 \right)}d\eta}}-\underset{+ln\left( \eta+1 \right)}{\underbrace{\int\frac{1}{\left( \eta+1 \right)}d\eta}} \right]$$

And the solution is:

$$=\frac{1}{2}\left[ \ln\left( \eta-1 \right)-\ln\left( \eta+1 \right) \right]=\frac{1}{2}\ln\left( \frac{\eta-1}{\eta+1} \right)$$

The overall expression:

$$\int\frac{1}{1-\frac{1}{\eta^{2}}}d\eta=\frac{1}{2}\ln\left( \frac{\eta-1}{\eta+1} \right)+\eta+C$$

The definite Integral:

$$\int_{\eta_{0}}^{\eta} \frac{1}{1-\frac{1}{\eta^{2}}}d\eta= \overset{@\eta}{\overbrace{\frac{1}{2}\ln\left( \frac{\eta-1}{\eta+1} \right)+\eta}}-\overset{@\eta_{0}}{\overbrace{\left[ \frac{1}{2}\ln\left( \frac{\eta_{o}-1}{\eta_{o}+1} \right)+\eta_{o} \right]}}$$

$$\int_{\eta_{0}}^{\eta} \frac{1}{1-\frac{1}{\eta^{2}}}d\eta=\frac{1}{2}\ln\left( \frac{\eta-1}{\eta+1}\cdot\frac{\eta_{o}+1}{\eta_{o}-1} \right)+\eta-\eta_{0}$$

Hence:

| $\frac{v_{ter}t}{y_{E}}=\eta-\eta_{0}+\frac{1}{2}\ln\left( \frac{\eta-1}{\eta+1}\cdot\frac{\eta_{o}+1}{\eta_{o}-1} \right)$ | (15) |
| --- | --- |

Were

$$\eta=\frac{y}{y_{E}}=\frac{y}{\sqrt{\frac{kq^{2}}{4mg}}} ; \eta_{0}=\frac{y_{0}}{y_{E}}=\frac{y_{0}}{\sqrt{\frac{kq^{2}}{4mg}}}$$

### Taylor Expansion O(5) for Charge Dominance

The full equation:

$$\frac{v_{ter}t}{y_{E}}=\eta-\eta_{0}+\frac{1}{2}\left[ \ln\left( 1-\eta\right)-\ln\left( 1+\eta\right) \right]-\frac{1}{2}[\ln\left( 1-\eta_{0} \right)-\ln\left( 1+\eta_{0} \right)]$$

Reorganizing:

$$2\frac{v_{ter}t}{y_{E}}=2\eta-2\eta_{0}+\left[ \ln\left( 1-\eta\right)-\ln\left( 1+\eta\right) \right]-[\ln\left( 1-\eta_{0} \right)-\ln\left( 1+\eta_{0} \right)]$$

$$2\frac{v_{ter}t}{y_{E}}=\underset{f(\eta)}{\underbrace{\left[ 2\eta+\ln\left( 1-\eta\right)-\ln\left( 1+\eta\right) \right]}}-\underset{f(\eta_{0})}{\underbrace{[2\eta_{0}+\ln\left( 1-\eta_{0} \right)-\ln\left( 1+\eta_{0} \right)]}}$$

We define the function:

$$f\left( \eta\right)=2\eta+\ln\left( 1-\eta\right)-\ln\left( 1+\eta\right)$$

Hence:

$$2\frac{v_{ter}t}{y_{E}}=f\left( \eta\right)-f\left( \eta_{0} \right)$$

Expanding a Taylor series for $\ln\left( 1+\eta\right)$ (around $\eta\to0$) for O(5):

$$\ln\left( 1+\eta\right)=\eta-\frac{\eta^{2}}{2}+\frac{\eta^{3}}{3}-\frac{\eta^{4}}{4}+\underset{O\left( 5 \right)}{\underbrace{\frac{\eta^{5}}{5}-\frac{\eta^{6}}{6}\cdot\cdot\cdot}}$$

Expanding a Taylor series for $\ln\left( 1-\eta\right)$ (around $\eta\to0$):

$$\ln\left( 1-\eta\right)=-\eta-\frac{\eta^{2}}{2}-\frac{\eta^{3}}{3}-\frac{\eta^{4}}{4}\underset{O(5)}{\underbrace{-\frac{\eta^{5}}{5}-\frac{\eta^{6}}{6}\cdot\cdot\cdot}}$$

The resulting expression for $f(\eta)$

$$f\left( \eta\right)=2\eta-\eta-\frac{\eta^{2}}{2}-\frac{\eta^{3}}{3}-\frac{\eta^{4}}{4}-\left( \eta-\frac{\eta^{2}}{2}+\frac{\eta^{3}}{3}-\frac{\eta^{4}}{4} \right)+O(5)$$

We note that all the even numbers are cancelled:

$$f\left( \eta\right)=2\eta-2\eta-\frac{\eta^{2}}{2}-\frac{\eta^{3}}{3}-\frac{\eta^{4}}{4}+\frac{\eta^{2}}{2}-\frac{\eta^{3}}{3}+\frac{\eta^{4}}{4}+O(5)$$

$$f\left( \eta\right)\approx-\frac{2}{3}\eta^{3}$$

In a similar manner, for $\eta_{0}:$

$$f\left( \eta_{0} \right)=-\frac{2}{3}\eta_{0}^{3}$$

Back to the full expression we get:

$$2\frac{v_{ter}t}{y_{E}}=f\left( \eta\right)-f\left( \eta_{0} \right)=-\frac{2}{3}\eta^{3}+\frac{2}{3}\eta_{0}^{3}$$

Dividing by 2:

$$\frac{v_{ter}t}{y_{E}}=\frac{\eta_{0}^{3}}{3}-\frac{\eta^{3}}{3}$$

Rearranging:

$$\eta^{3}=\eta_{0}^{3}-\frac{3v_{ter}t}{y_{E}}$$

We recall:

$$\eta=\frac{y}{y_{E}}=\frac{y}{\sqrt{\frac{kq^{2}}{4mg}}} ; \eta_{0}=\frac{y_{0}}{y_{E}}=\frac{y_{0}}{\sqrt{\frac{kq^{2}}{4mg}}}$$

Therefore:

$$y=\left( y_{0}^{3}-\frac{3}{4}\frac{v_{ter}\cdot kq^{2}t}{mg} \right)^{\frac{1}{3}}$$

We recall also that: $v_{ter}=\tau g=Bmg$

$$y=\left( y_{0}^{3}-\frac{3}{4}\frac{Bmg\cdot kq^{2}t}{mg} \right)^{\frac{1}{3}}$$

And the final expression we get

| $y=\left( y_{0}^{3}-\frac{3}{4}B\cdot kq^{2}t \right)^{\frac{1}{3}}$ | (16) |
| --- | --- |

Demanding the condition $y\left( t_{f} \right)=0$:

$$t_{f}=\frac{4y_{0}^{3}}{3k_{e}Bq^{2}} \to y_{0}=\left( \frac{3k_{e}Bq^{2}t_{f}}{4} \right)^{\frac{1}{3}}$$

A non-dimensional presentation can be made using $t_{f}$ as a scaling parameter:

$$0<t^{'}=\frac{t}{t_{f}}<1$$

Rearranging:

$$y=\left( y_{0}^{3}-\frac{y_{0}^{3}}{y_{0}^{3}}\frac{3}{4} k_{e}Bq^{2}\cdot t \right)^{1/3}$$

We can get the non-dimensional time:

$$y=y_{0}\left( 1+t^{'} \right)^{1/3}$$

Dividing by the initial height $y_{0}$ will result in non-dimensional height: $y^{'}=\frac{y}{y_{0}}$ analogues to $\eta_{0}$:

| $y'=\left( 1+t^{'} \right)^{1/3}$ | (17) |
| --- | --- |

### Energy Conservation derivation

A factor limiting the speed of a particle is the particle’s mechanical mobility $B$ within the air. The drag force acting on the particle is proportional to the velocity gained due to electrostatic attraction, counteracting and reducing its acceleration

To validate the time scale estimates obtained from both the analytical and numerical approaches, we derived an analytical solution based on the energy equation. To simplify the derivation, we considered a limiting case where viscous drag is neglected, allowing us to examine whether deposition time significantly decreases in the absence of resistance. We began with the general case applicable to all scenarios and progressively simplified it to isolate the role of the drag force in determining deposition time. Gravitational potential Energy $U_{g}$ define as:

$$-\nabla U_{g}=\vec{F}_{g}$$

We recall we are solving a case we gravity opposed the tissue in the (+$\hat{y})$:

$$-\frac{dU_{g}}{dy}=+mg$$

$$dU_{g}=-mg dy$$

$$\int_{i}^{f} dU_{g}=\int_{h_{0}}^{y} -mg dy$$

$$U_{f}-U_{i}=-mg(y-h_{0})$$

Hence, The gravitational potential energy is:

| $U_{g}(y)=-mgy$ | (18) |
| --- | --- |

$$U_{g}(h_{0})=-mgh_{0}$$

In a similar manner, the electric force acts on a particle: $\vec{F}_{e}=\frac{k_{e}q^{2}}{4y^{2}} (-\hat{y})$, hence the electric potential energy $U_{e}$ can be defined by:

$$-\frac{dU_{e}}{dy}=-\frac{k_{e}q^{2}}{4y^{2}}$$

Rearranging:

$$dU_{e}=\frac{k_{e}q^{2}}{4y^{2}}dy$$

Performing integration on both sides:

$$\int_{i}^{f} dU_{e}=\int_{h_{0}}^{y} \frac{k_{e}q^{2}}{4}y^{-2}dy$$

$$U_{f}-U_{i}=\frac{k_{e}q^{2}}{4}\left[ \frac{-1}{y} \right]_{h_{0}}^{y}$$

$$U_{f}-U_{i}=-\frac{k_{e}q^{2}}{4}\left[ \frac{1}{y} \right]_{h_{0}}^{y}$$

$$U_{f}-U_{i}=\left[ -\frac{k_{e}q^{2}}{4y} \right]-\left[ -\frac{k_{e}q^{2}}{4h_{0}} \right]$$

We get, the electric potential energy $U_{e}$:

| $U_{e}\left( y \right)=-\frac{k_{e}q^{2}}{4y}$ | (19) |
| --- | --- |

And the initial value is

$$U_{e}\left( h_{0} \right)=-\frac{k_{E}q^{2}}{4h_{0}}$$

The total energy, including the kinetic energy:

$$E_{total}=E_{k}+U\left( y \right)=\frac{1}{2}mv^{2}-\frac{k_{e}q^{2}}{4y}-mgy$$

Using conservation of energy

$$E_{0}=E\left( y \right)$$

Considering all energies involved:

| $\frac{1}{2}mv_{0}^{2}-\frac{k_{e}q^{2}}{4h_{0}}-mgh_{0}=\frac{1}{2}mv^{2}-\frac{k_{e}q^{2}}{4y}-mgy-W_{friction}$ | (20) |
| --- | --- |

Where $W_{friction}$ is the energy loss due to the air resistance:

$$W_{friction}=\int_{h_{0}}^{y} F_{drag} dy \int_{h_{0}}^{y} \left[ -\frac{v_{\left( y \right)}}{B} \right]dy$$

$$\frac{1}{2}mv_{0}^{2}-\frac{k_{e}q^{2}}{4h_{0}}-mgh_{0}=\frac{1}{2}mv^{2}-\frac{k_{e}q^{2}}{4y}-mgy-\underset{W_{friction}\approx0}{\underbrace{\int_{h_{0}}^{y} \left[ -\frac{v_{\left( y \right)}}{B} \right]dy}}$$

Assuming no friction loss via heat dissipation of drag force (very large *B*) we get the mechanical energy conservation since all forces are conservative:

| $\frac{1}{2}mv_{0}^{2}-\frac{k_{e}q^{2}}{4h_{0}}-mgh_{0}=\frac{1}{2}mv^{2}-\frac{k_{e}q^{2}}{4y}-mgy$ | (21) |
| --- | --- |

Considering initial velocity to be neglectable $v_{0}=0$

$$\frac{1}{2}mv^{2}=-\frac{k_{e}q^{2}}{4h_{0}}+\frac{k_{e}q^{2}}{4y}-mgh_{0}+mgy$$

$$v=\pm\sqrt{\frac{k_{e}q^{2}}{2m}\left( \frac{1}{y}-\frac{1}{h_{0}} \right)+2g\left( y-h_{0} \right)}$$

Neglecting Gravity (very small mass and very short distances) the velocity is:

$$v=\pm\sqrt{\frac{k_{e}q^{2}}{2m}\left( \frac{1}{y}-\frac{1}{h_{0}} \right)}$$

Since the particle approaches the tissue ($y<h_{0}$) we take the negative sign solution:

| $v=-\sqrt{\frac{k_{e}q^{2}}{2m}\left( \frac{1}{y}-\frac{1}{h_{0}} \right)}$ | (22) |
| --- | --- |

For replacement we can write displacement as:

$$\frac{dy}{dt}=-\sqrt{\frac{kq^{2}}{2m}}\sqrt{\left( \frac{1}{y}-\frac{1}{h_{0}} \right)}$$

Reorganizing the expression and applying integration (implementing separation of variables)

$$\int_{h_{0}}^{y(t)} \left[ \frac{1}{y}-\frac{1}{h_{o}} \right]^{-\frac{1}{2}}dy=-\int_{0}^{t} \sqrt{\frac{k_{e}q^{2}}{2m}}dt$$

Organize:

$$\int_{h_{0}}^{y(t)} \left[ \underset{=z_{\left( t \right)}}{\underbrace{\frac{1}{y_{\left( t \right)}}-\frac{1}{h_{o}}}} \right]^{-\frac{1}{2}}dy=-t\sqrt{\frac{k_{e}q^{2}}{2m}}$$

Defining a new parameter “z”

$$z=\frac{1}{y}-\frac{1}{h_{o}} \to y=\frac{1}{z+\frac{1}{h_{0}}}$$

$$\frac{dy}{dz}=\frac{d}{dz}\left( y^{-1}-\frac{1}{h_{0}} \right)=-y^{-2}$$

$$dz=-\frac{1}{y^{2}}dy$$

$$dy=-y^{2}dz$$

Therefore:

$$dy=-\frac{dz}{\left( z+\frac{1}{h_{0}} \right)^{2}}$$

Hence the integral is:

$$-\int_{z_{0}}^{z(t)} \frac{dz}{\sqrt{z}\left( z+\frac{1}{h_{0}} \right)^{2}}=?$$

Were $z=z(y)=\frac{1}{y}-\frac{1}{h_{o}}$ and $z_{0}=z\left( y=h_{0} \right)=\frac{1}{h_{0}}-\frac{1}{h_{o}}=0$

The integral solution:

$$-\int_{0}^{z} \frac{dz}{\sqrt{z}\left( z+\frac{1}{h_{0}} \right)^{2}}={-\left[ \frac{h^{2}\sqrt{z}}{hz+1}+h^{1.5}\arctan\left( \sqrt{hz} \right) \right]}_{0}^{z}$$

Substitute boundaries:

$${-\left[ \frac{h^{2}\sqrt{z}}{hz+1}+h^{1.5}\arctan\left( \sqrt{hz} \right) \right]}_{0}^{z}=-\frac{(h_{0}^{2.5} z+h_{0}^{1.5})\arctan\left( \sqrt{hz} \right)+ h_{0}^{2} \sqrt{z}}{h_{0}z + 1}$$

Back to the original equation:

$$\frac{(h_{0}^{2.5}z+h_{0}^{1.5})\arctan\left( \sqrt{h_{0}z} \right)+h_{0}^{2}\sqrt{z}}{h_{0}z + 1}= \int_{0}^{t} \sqrt{\frac{kq^{2}}{2m}}dt$$

Substitute $z=\frac{1}{y}-\frac{1}{h_{0}}$

$$\frac{\left[ h_{0}^{2.5}\left( \frac{1}{y}-\frac{1}{h_{o}} \right)+h_{0}^{1.5} \right]\arctan\sqrt{\frac{h_{0}}{y}-1}+ h_{0}^{2}\sqrt{\frac{1}{y}-\frac{1}{h_{o}}}}{h_{0}\left( \frac{1}{y}-\frac{1}{h_{o}} \right) + 1}$$

We take multiply each ($\frac{1}{y}-\frac{1}{h_{0}}$) by $h_{0}$

$$\frac{\left[ h_{0}^{1.5}\left( \frac{h_{0}}{y}-1 \right)+h_{0}^{1.5} \right]\arctan\sqrt{\frac{h_{0}}{y}-1}+ h_{0}^{1.5} \sqrt{\frac{h_{0}}{y}-1}}{\frac{h_{0}}{y}-1 + 1}=$$

$$h_{0}^{1.5}\frac{\left[ \left( \frac{h_{0}}{y}-1 \right)+1 \right]\arctan\sqrt{\frac{h_{0}}{y}-1}+\sqrt{\frac{h_{0}}{y}-1}}{\frac{h_{0}}{y}}=$$

$$yh_{0}^{0.5}\left\{ \frac{h_{0}}{y}\arctan\sqrt{\frac{h_{0}}{y}-1} + \sqrt{\frac{h_{0}}{y}-1} \right\}=$$

$$h_{0}^{1.5}\arctan\sqrt{\frac{h_{0}}{y}-1} + h_{0}^{0.5} y\sqrt{\frac{h_{0}}{y}-1}=$$

Back into the main equation:

| $h_{0}^{0.5} y\sqrt{\frac{h_{0}}{y}-1} + h_{0}^{1.5}\arctan\sqrt{\frac{h_{0}}{y}-1} =t\sqrt{\frac{k_{e}q^{2}}{2m}}$ | (23) |
| --- | --- |

This is the govern equation for the particle dynamic under electrostatic force only (no gravity, diffusion and drag). For the case of $y\to0$ we are approaching the final time $t_{f}$ for deposition:

$$\underset{\to\sqrt{y} \to0}{\underbrace{h_{0}^{0.5}y\sqrt{\left( \frac{h_{0}}{y}-1 \right)}}}+ h_{0}^{1.5}\underset{\to\frac{\pi}{2}}{\underbrace{\arctan\left[ \sqrt{\frac{h_{0}}{y}-1} \right]}} =t\sqrt{\frac{k_{e}q^{2}}{2m}}$$

$$\frac{\pi}{2}h^{1.5}=t_{f}\sqrt{\frac{k_{e}q^{2}}{2m}}$$

Rearranging the terms:

| $t_{f}=\frac{\pi}{2q}\sqrt{\frac{2mh^{3}}{k_{e}}}$ | (24) |
| --- | --- |

Recalling we are dealing with a spherical particle $m=\rho\frac{\pi d_{p}^{3}}{6}$ We would get:

| $t_{f}=\frac{1}{q}\sqrt{\frac{\rho\left( \pi d_{p}h \right)^{3}}{12k_{e}}}$ | (25) |
| --- | --- |

Calculation of the charge cases presented in the paper are supply below:

| $q$[e] | $d_{p} [\mu m]$ | $t_{f}=\frac{1}{q}\sqrt{\frac{\rho\left( \pi d_{p}h \right)^{3}}{12k_{e}}}$ | $t_{f}$including drag and gravity (theory and simulation) |
| --- | --- | --- | --- |
| $415$ | $0.2$ | $79.88 \mu s$ | << 76 ms |
| $135$ |  | $0.24$ms | << 0.151 s |
| $650$ | $2$ | $16$ ms | << 0.195 s |

Table S2: Time deposition in the absence of gravity and drag compared to

Our solution predicts an airborne time that is more than two orders of magnitude longer than that of the diffusive particle which includes drag, further emphasizing again the critical role of mechanical mobility *B*, particularly due to particle shape, in counteracting electrostatic forces.

### Stopping Distance After Deposition

Charge particles rapidly accelerate toward the tissue, reaching high velocities. This phenomenon was first captured on video in a previous study and was hypothesized to result from electrostatic attraction [3]. The rapid acceleration clearly stems from the inverse square dependency of the electrostatic force *F_e_ ∝ y^−2^*. As a particle moves closer to the tissue, the electrostatic field strengthens, increasing its acceleration toward the surface and creating a feedback loop that further amplifies its velocity. For the 2 µm particles, our numerical simulation and allows us to compare the final velocity $v_{e}$ (~ 12 cm/s) in the charged case (Figure S4b) to the terminal velocity achieved in free fall scenario $v_{ter}=\tau_{p}g$ (~ 138 µm/s, Figure S4d). $\frac{v_{e}}{\tau g}=1445.78 \sim{10}^{3}.$The results show an immense difference of more than three orders of magnitude, highlighting the strong influence of electrostatic forces near the tissue.
From a fluid mechanics perspective, when considering particle interaction with mucus following deposition, despite the significant differences in velocity, the viscosity of mucus is so high (μ≈0.1 Pa·s) resulting in a very short stopping distances for both cases:

Relaxation time in mucus:

$$\tau_{\mu}=\frac{\rho_{p}d_{p}^{2}}{18\mu_{mucus}}=2.33\times{10}^{-9} [s]$$

The stopping distance in every case is presented in the table below, where the mucus thickness was approximated to be ~ $70\mu m$

| Size $d_{p}[\mu m]$ | Charged | Neutralized case $q\sim1e$ | Velocity Ratio $\frac{v_{e}}{v_{other}}$ | Distance Ratio  $\frac{y_{stop}^{charge}}{70\mu m}=?$ |
| --- | --- | --- | --- | --- |
| $2$ | $q\sim650 e$ $v_{e}=12 cm/s$  $y_{stop}=v_{e} \tau_{\mu}$ $y_{stop}=2.8\times{10}^{-10}$ | $v_{ter}=\tau g=83 \mu\frac{m}{s}$ $y_{stop}=v_{ter} \tau_{\mu}*\tau_{\mu}$ $y_{stop}=1.93\times{10}^{-13}$ | $\sim{10}^{3}$ | $\frac{2.8\times{10}^{-10}}{70\times{10}^{-6}} \sim{10}^{-6}$ |
| $0.2$ | $q\sim415 e$ $v_{e}=1.00 m/s$  $y_{stop}=v_{e} \tau_{\mu}$  $y_{stop}=2.33\times{10}^{-9}$ | $v_{diff}\approx\frac{\Delta_{y}}{\Delta t}=\sqrt{\frac{2D}{\Delta t}}= 0.02$  $y_{stop}=v_{diff} \tau_{\mu}$  $y_{stop=}=5.03\times{10}^{-11}$ | $\sim50$ | $\frac{2.33\times{10}^{-9}}{70\times{10}^{-6}}\sim{10}^{-5}$ |

Table S3: Stopping distance and final velocity comparison

We can clearly see that although the velocity next to the tissue is very height in the charge case the stopping distance takes place immediately mainly due to the mucus viscosity.

In different experiment [50], although the experimental conditions differed from classical image charge setups, similar electrostatically driven particle accelerations were captured in video provided in the supplementary, of PDMS model with aerosols at the lung-relevant scale, demonstrating the strong ability of electrostatic forces to induce high particle velocities. In the referenced study, it is likely that the PDMS material acquired a surface charge *(q₁)* through handling, while the aerosols carried a separate charge (*q₂*) generated during aerosolization. The resulting attraction force observed in the Supplementary Video qualitatively follows the expected electrostatic behavior, with *F_e_​∝ q_1_​q_2_*​. Conceptually, this situation is analogous to an image charge phenomenon, where the effective aerosol charge could be approximated as $q=\sqrt{q_{1}q_{2}}$, and the model would act as a neutral conductive surface facilitating the electrostatic interaction.

## Deposition Fraction

The graph below (Fig. S4) presents deposition data for two cases not included in the main manuscript: **0.5 μm particles**, which showed no significant change in deposition and were therefore excluded from the main discussion, and 2 μm particles exposed under conditions where gravity was aligned with the airway geometry. The latter serves as a sanity check to validate gravitational sedimentation as a deposition mechanism. As shown, it is difficult to distinguish between the deposition patterns, indicating that both electrostatic and gravitational forces contribute to particle behavior. Notably, although the exposure time in the charged case is approximately half that of the neutralized case, sufficient deposition is still achieved, suggesting a synergistic effect between the two mechanisms.


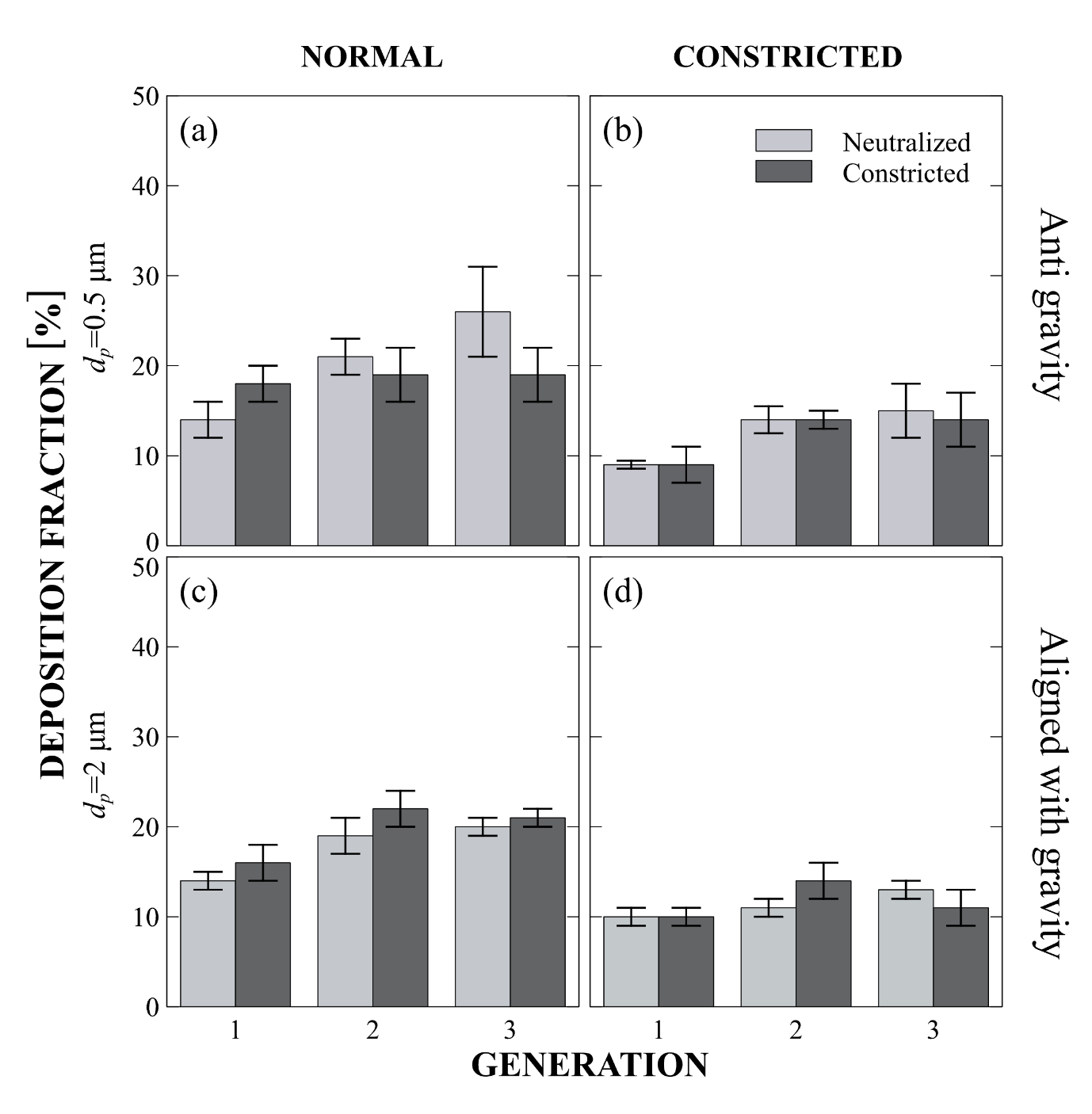


Figure S4: Deposition Fractions (DF) across airway generations in the in vitro model. The x-axis indicates the airway generation, while the y-axis represents the corresponding DF. Rows distinguish between particle sizes (0.5 µm anti-gravity and 2 µm with gravity direction), and columns represent airway conditions (normal vs. constricted). Light gray bars denote neutralized particle exposures, while dark gray bars indicate charged particle exposures.

## Statistical Analysis

Attached is a table of the exact p-values and Chi squared value obtained in the statistical analysis mentioned in the paper.

| Response: **Deposition Fraction** | Chi squared | Df | Pr(>Chi squared) | Significance |
| --- | --- | --- | --- | --- |
| Intercept | 45.7130 | $1$ | $1.369\times{10}^{-11}$ | *** |
| $d_{p}$ | 36.7540 | 2 | $1.045\times{10}^{-8}$ | *** |
| $q$ | 52.1450 | 1 | $5.155\times{10}^{-13}$ | *** |
| Gen | $4.6914$ | 2 | $0.09578$ | . |
| State | $111.5452$ | $1$ | $<2.2\times{10}^{-16}$ | *** |
| $d_{p}$:$q$ | $39.1775$ | $2$ | $3.110\times{10}^{-9}$ | *** |
| $d_{p}$:Gen | $10.2473$ | $4$ | $0.03646$ | * |
| $q$:Gen | $66.7955$ | $2$ | $3.130\times{10}^{-15}$ | *** |
| $d_{p}$:$q$:Gen | $26.4829$ | $4$ | $2.529\times{10}^{-5}$ | *** |

Table S4 type III p-values and Chi squared value obtained in the statistical analysis

| term | estimate ($\beta$) | SE | 95%LCL | 95%UCL | p.value |
| --- | --- | --- | --- | --- | --- |
| (Intercept) | $10\%$ | $1\%$ | $7\%$ | $13\%$ | $1.55\times{10}^{-10}$ |
| $d_{p}0.5$ | $-2\%$ | $2\%$ | $-6\%$ | $2\%$ | $0.333$ |
| $d_{p}2$ | $-14\%$ | $2\%$ | $-18\%$ | $-9\%$ | $2.301\times{10}^{-8}$ |
| Charged | $15\%$ | $2\%$ | $11\%$ | $19\%$ | $1.127\times{10}^{-11}$ |
| G2 | $4\%$ | $2\%$ | $0\%$ | $8\%$ | $0.032$ |
| G3 | $2\%$ | $2\%$ | $-1\%$ | $6\%$ | $0.223$ |
| Normal State | $8\%$ | $1\%$ | $6\%$ | $9\%$ | $6.56\times{10}^{-21}$ |
| $d_{p}$0.5: Charged | $-13\%$ | $3\%$ | $-18\%$ | $-7\%$ | $1.077\times{10}^{-5}$ |
| $d_{p}$2: Charged | $6\%$ | $3\%$ | $-1\%$ | $13\%$ | $0.071$ |
| $d_{p}$0.5:G2 | $2\%$ | $3\%$ | $-4\%$ | $7\%$ | $0.594$ |
| $d_{p}$2:G2 | $-4\%$ | $3\%$ | $-11\%$ | $2\%$ | $0.194$ |
| $d_{p}$0.5:G3 | $7\%$ | $3\%$ | $1\%$ | $12\%$ | $0.022$ |
| $d_{p}$2:G3 | $-2\%$ | $3\%$ | $-9\%$ | $4\%$ | $0.461$ |
| Charged:G2 | $-18\%$ | $3\%$ | $-24\%$ | $-13\%$ | $1.77\times{10}^{-9}$ |
| Charged:G3 | $-22\%$ | $3\%$ | $-28\%$ | $-17\%$ | $9.031\times{10}^{-13}$ |
| $d_{p}$0.5: Charged:G2 | $17\%$ | $4\%$ | $9\%$ | $24\%$ | $5.041\times{10}^{-5}$ |
| $d_{p}$2: Charged:G2 | $15\%$ | $5\%$ | $5\%$ | $24\%$ | $0.002$ |
| $d_{p}$0.5: Charged:G3 | $18\%$ | $4\%$ | $10\%$ | $26\%$ | $1.582\times{10}^{-5}$ |
| $d_{p}$2: Charged:G3 | $13\%$ | $5\%$ | $4\%$ | $23\%$ | $0.006$ |

Table S5: Regression results showing Beta estimates for particle size, charge, device generation, and their interactions. Positive or negative Beta values indicate the strength and direction of each effect on aerosol behavior.

| contrast | $d_{p} [ \mu m]$ | Gen | estimate (beta) | SE | p. value | Significance |
| --- | --- | --- | --- | --- | --- | --- |
| Q100 - Q1 | 0.2 | G1 | 14.93% | 0.0207 | $2.10\times{10}^{-11}$ | *** |
|  |  | G2 | -3.52% |  | $0.090$ | . |
|  |  | G3 | -7.42% |  | $4.46\times{10}^{-4}$ | *** |
|  | 0.5 | G1 | 2.17% | 0.0192 | $0.261$ | . |
|  |  | G2 | 0.26% |  | $0.893$ | . |
|  |  | G3 | -2.51% |  | $0.194$ | . |
|  | 2 | G1 | 21.00% | 0.0263 | $2.70\times{10}^{-13}$ | *** |
|  |  | G2 | 17.13% |  | $9.29\times{10}^{-10}$ | *** |
|  |  | G3 | 11.87% |  | $1.22\times{10}^{-5}$ | *** |

*Table S6: Summary of estimated differences in Deposition Fraction (DF) between charged (Q100) and neutralized (Q1) aerosols across particle sizes and airway generations. The table includes the estimate (%), standard error (SE), and corresponding p-values*

## MATLAB Simulation for Particle Dynamics

In this section, we describe the procedure used to obtain the motion dynamics of individual particles. As an illustrative example, Figure S5 shows a 0.5 μm particle depositing within the constricted airway region, while under identical conditions, the same particle escapes through the normal-sized airway. This highlights the sensitivity of particle trajectories to geometric variations in the airway structure.


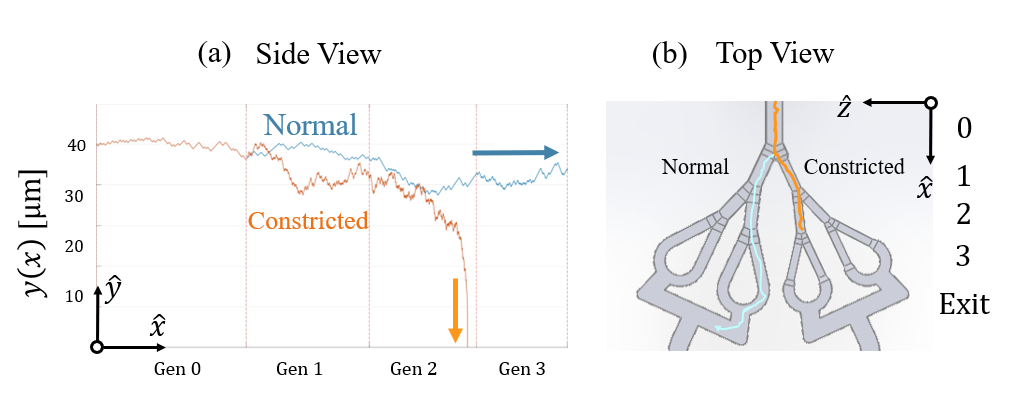


Figure S5: Numerical simulation of a 0.5 μm diameter particle charged with 220e, released from a height corresponding to 10% of the diameter of generation 0 (G0). Two scenarios are presented: an orange trajectory showing the particle depositing at the end of generation 2 within the constricted airway, and a blue trajectory showing the particle escaping the model through the normal non-constricted) airway. (a) Side view in the XY plane, illustrating the actual particle paths. (b) Top-down view provided for spatial orientation purposes only—not representing the true particle trajectory.

In this section, we outline the procedure for modeling particle dynamics in both time and space using MATLAB software, as illustrated in Figure S5. To compute the particle trajectory, we employed Finite-Difference Formulas for the second derivative in the governing equation.

### Theory

It's important to note that for particles moving along the flow in the x-direction, the altitude of the particle is represented on the Y-axis. According to Newton's second law, the electrostatic force acts perpendicular to the surface and in conjunction with gravity and drag forces (diffusion will be incorporated at a later stage).

| $F_{e}+F_{d}+F_{g}=ma_{y}$ | **(**26) |
| --- | --- |

Substitute forces resulted in a non-linear second order ODE.

| $-\frac{k_{e}q^{2}}{4{y_{\left( t \right)}}^{2}}-\frac{1}{B}{v_{y}}_{\left( t \right)}-mg=m{a_{y}}_{(t)}$ | **(**27) |
| --- | --- |

Please note that the sign of the drag force determines from the sign of the velocity as it is an opposing force. We use backward difference method (Explicit Euler Method) as follows, and calculate the velocity based on one initial condition:

| $v_{y}(t)=\lim_{dt\to0} \frac{y_{\left( t \right)}-y_{\left( t-dt \right)}}{dt}\approx\frac{y_{\left( t \right)}-y_{\left( t-\Delta t \right)}}{\Delta t}$ | **(**28) |
| --- | --- |

And for acceleration in a similar manner:

| ${a_{y}}_{(t)}=\lim_{dt\to0} \frac{v_{\left( t \right)}-v_{\left( t-dt \right)}}{dt}\approx\frac{1}{\Delta t}\left[ \frac{y_{\left( t \right)}-y_{\left( t-\Delta t \right)}}{\Delta t}-\frac{y_{\left( t-\Delta t \right)}-y_{\left( t-2\Delta t \right)}}{\Delta t} \right]$ | **(**29) |
| --- | --- |

Considering $d\left( dt \right)\to0$, we get an expression for the acceleration using:

| ${a_{y}}_{(t)}=\frac{y_{\left( t \right)}-2y_{\left( t-\Delta t \right)}+y_{\left( t-2\Delta t \right)}}{{\Delta t}^{2}}$ | **(**30) |
| --- | --- |

Where the initial conditions are the initial height: $y_{\left( t=0 \right)}=y_{\left( t=\Delta t \right)}=y_{\left( t=2\Delta t \right)}=y_{i=1}=y_{i=2}$

Summing up all, we end up with an equation of $y(t)$ :

| $-\frac{kq^{2}}{4y_{\left( t \right)}^{2}}-\frac{1}{B}\left( \frac{y_{\left( t \right)}-y_{\left( t-\Delta t \right)}}{\Delta t} \right)-mg=m\left( \frac{y_{\left( t \right)}-2y_{\left( t-\Delta t \right)}+y_{\left( t-2\Delta t \right)}}{\Delta t^{2}} \right)$ | **(**31) |
| --- | --- |

Multiplying by “$4By_{\left( t \right)}^{2}dt^{2}"$, and applying $\tau=Bm$, we then get a cubic polynomial equation:

| $y^{3}\underset{C_{3}}{\underbrace{\left( 4\tau+4\Delta t \right)}}+y^{2}\underset{C_{2}}{\underbrace{\left[ 4\tau y_{\left( t-2\Delta t \right)}-\left( 4dt+8\tau\right)y_{\left( t-\Delta t \right)}+4\tau g{\Delta t}^{2} \right]}}+\underset{C_{0}}{\underbrace{Bkq^{2}{\Delta t}^{2}}}=0$ | **(**32) |
| --- | --- |

For simplicity, we write:

| $C_{3}y^{3}+C_{2}y^{2}+C_{0}=0$ | **(**33) |
| --- | --- |

(Note that the coefficient for $y^{1}$ is zero since the expression is absent in the equation, hence $C_{1}=0)$.

We then numerically compute this equation using MATLAB, disregarding any unphysical solutions (nor real). Once we identify the correct solution, we update the results by adding a randomly determined step size (∆) representing Brownian motion, achieved through particle diffusion.

| $y_{\left( t \right)}=y_{\left( t \right)}\pm\Delta_{y}$ | **(**34) |
| --- | --- |

Where the random step size (∆) is of magnitude:

| $\Delta=\sqrt{KD_{mol}\Delta t}=\sqrt{K\underset{D_{mol}}{\underbrace{\frac{k_{B}TC_{c}}{3\pi\mu_{f}d_{p}}}}\Delta t}$ | **(**35) |
| --- | --- |

Here, K=2 for 1D motion along the y-axis. It is important to note that even though the final result is in 2D, we solve each axis separately and combined them into a single plot. Therefore, the 1D constraint should be considered.

Along X-axis we need to consider the velocity profile and the velocity of the particle for each iteration. Our govern equation for the advance of the particle in X-axis is:

| $x_{\left( t \right)}=x_{\left( t-dt \right)}+u_{\left( t \right)}dt\pm\Delta_{x}$ | **(**36) |
| --- | --- |

The velocity is calculated using a developed Poiseuille flow profile in rectangular ducts (as mentioned in Shah and London, laminar Flow Forced Convection in Ducts, 1978, Eqn 332-338).

### Velocity profile in Rectangular Ducts


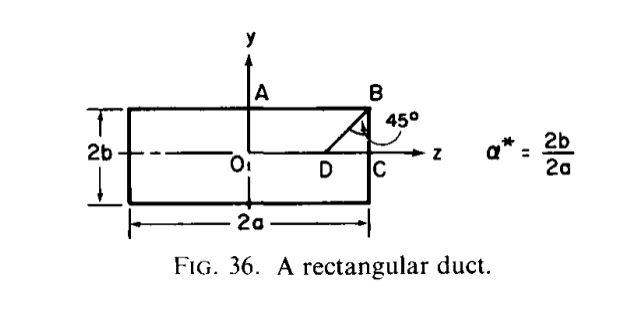
The velocity is calculated using a developed Poiseuille flow profile in rectangular ducts [as mentioned in Shah and London, laminar Flow Forced Convection in Ducts, 1978, Eqn 332-338)]. For a general rectangular channel, the flow is in the x direction and for a cross section:

Figure S6: Schematic channel cross-section

|  | $u\left( y,z \right)= -\frac{16Ca^{2}}{\pi^{3}}\sum_{n=1,3,...}^{\infty} \frac{1}{n^{3}}\left( -1 \right)^{0.5\left( n-1 \right)}\left[ 1-\frac{\cosh(n\pi y/2a)}{\cosh(n\pi b/2a)} \right]\cos\left( \frac{n\pi z}{2a} \right)$ | **(**37) |
| --- | --- | --- |

Where C can be determined through the flowrate and the average velocity, as given by:

|  | $u_{ave}\left( y,z \right)= -\frac{Ca^{2}}{3}\left[ 1-\frac{192}{\pi^{5}}\left( \frac{a}{b} \right)\sum_{n=1,3,...}^{\infty} \frac{1}{n^{3}}\tanh\left( \frac{\pi nb}{2a} \right) \right]$ | **(**38) |
| --- | --- | --- |

For our squared channel $2a=2b=D$ :

|  | $u\left( y,z \right)= -\frac{4CD^{2}}{\pi^{3}}\sum_{n=1,3,...}^{\infty} \frac{1}{n^{3}}\left( -1 \right)^{0.5\left( n-1 \right)}\left[ 1-\frac{\cosh(n\pi y/D)}{\cosh(n\pi/2)} \right]\cos\left( \frac{n\pi z}{D} \right)$ | **(**39) |
| --- | --- | --- |

Hence:

|  | $u_{ave}\left( y,z \right)= -\frac{CD^{2}}{12}\left[ 1-\frac{192}{\pi^{5}}\sum_{n=1,3,...}^{\infty} \frac{1}{n^{3}}\tanh\left( \frac{\pi n}{2} \right) \right]$ | **(**40) |
| --- | --- | --- |

The result for the velocity flow profile is presented in Figure S7.


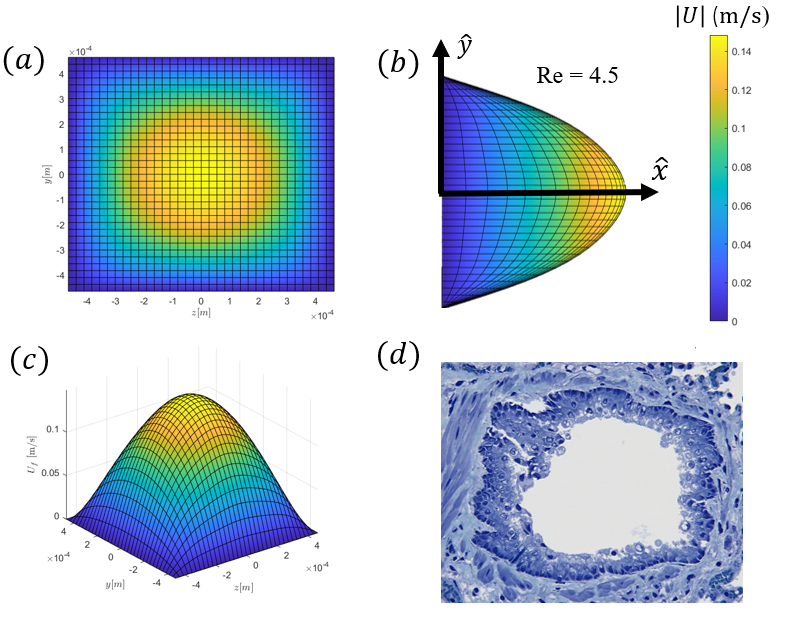


Figure S7: Velocity Magnitude Analysis, Reynolds number (Re = 4.5) characteristic of the terminal bronchiole, Laminar Hagen–Poiseuille Flow was adapted to Generation 11. (a) Velocity magnitude in the YZ plane. (b) Velocity magnitude in the XY plane. (c) 3D view of velocity magnitude. (d) Real lung cross-section illustrating that in the bronchiole region, airways are not perfect circles as simplified by the Weibel model, but rather have arbitrary shapes. (Courtesy of Professor Carsten Ehrhardt, Trinity College Dublin, Ireland)

### Initial height and characteristic length

We defined the characteristic length $L_{c}$ for the electrostatic force and the Brownian diffusion to be the distance from the tissue toward the airborne particle. When observing a particle with a high chance of being deposited, it must be found at some point near the tissue. We used as $L_{c}=0.05R_{0}=0.025D_{0}$, where the radius $R_{0}$ of the inlet generation was based on average human adult lungs [18]. We chose this characteristic for two reasons: (i) first, this small proximity to the tissue enables us to simplify the electric interaction between the tissue and the airborne particle using an image of charge method for tissue to act as a semi-infinite equipotential conducting surface. (ii) Secondly, this value corresponds to a significant enough portion of the lumen volume to alter deposition, 10% of $V_{tot}^{Cylinder}$ the total volume of the ideal cylindrical airway (derivation below). For the same reasons we chose this height as our initial condition for our simulations.

$$D$$

$$L_{C}$$

$$V_{shell}$$

$$d$$

Figure S8: Schematic cross-sectional view of the geometry: cylindrical airway or sphere representing the acinar region cavity. The total volume geometry V_tot_ has a diameter D, with d denoting the diameter of the inner volume V_in_. The inner volume is separated from the tissue by a distance L_c_.

#### Mathematical derivations

Determining the characteristic length, $L_{c}$, at which the influence of electrical forces relative to the diameter becomes significant depends strongly on the geometry of a cylindrical airway. For a general case let's define $X$, the ratio between the volume region prone to electrostatic $V_{shell}$ to the total volume of the geometry $V_{tot}$ (see Fig. S2).

|  | $X= \frac{V_{shell}}{V_{tot}}$ | (41) |
| --- | --- | --- |

Where the internal volume $V_{in}$ is the subtraction between them:

|  | $V_{in}=V_{tot}-V_{shell}$ | (42) |
| --- | --- | --- |

Now let’s derive $X$for any $L_{c}$ that has been chosen to represent the electrostatic force for both consider geometries.

$$X= \frac{V_{shell}}{V_{tot}}= \frac{L}{L}\frac{A_{ring}}{A_{tot}}= \frac{A_{tot}-A_{in}}{A_{tot}}=\frac{\left( \frac{\pi D^{2}}{4} \right)-\left( \frac{\pi d^{2}}{4} \right)}{\left( \frac{\pi D^{2}}{4} \right)}= 1- \frac{d^{2}}{D^{2}}$$

|  | $X=1- \frac{d^{2}}{D^{2}}$ | **(**43) |
| --- | --- | --- |

Note that $d = D - 2L_{c}$, therefore:

$$X=1-\frac{\left( D-2L_{c} \right)^{2}}{D^{2}}$$

$$4L_{c}^{2}-4DL_{c}+XD^{2}=0$$

By solving the quadratic equation, we get:

$$L_{c}=\frac{-\left( -4D \right)\pm\sqrt{\left( 4D \right)^{2}-4*4*\left( XD^{2} \right)}}{2*4}=\frac{4D\pm\sqrt{16D^{2}-16XD^{2}}}{8}$$

$$L_{c}=\frac{4D\pm4D\sqrt{1-X}}{8}= 0.5*\left( 1\pm\sqrt{1-X} \right)*D$$

We have two solutions:

|  | $L_{c_{\pm}}= 0.5*\left( 1\pm\sqrt{1-X} \right)D$ | **(**44) |
| --- | --- | --- |

Note the physical solution is one that must be satisfied by: $L_{c}<0.5D$

For $X_{Cylinder}$calculation for a given $L_{c}$ :

|  | $X_{Cylinder}={1-\left( \frac{L_{c}}{R}-1 \right)}^{2}$ | **(**45) |
| --- | --- | --- |

When we apply $L_{c}=0.05R$, we get $X_{Cylinder}=10\%$ where $X= \frac{V_{shell}}{V_{tot}}$.

### Error Calculation and Conversion

We recall breathing cycle takes approximately 4 seconds; thus, we aimed to achieve a significantly shorter time interval for the simulations. We chose an interval time between each MATLAB iteration of $dt =0.1$ millisecond, which is four orders of magnitude smaller than the breathing cycle. Alternatively, one might consider using the particle relaxation time, approximately one microsecond for inhaled aerosols, but this would require substantially more computational resources. Therefore, we verified that our chosen interval provides an optimal balance for numerical simulations, allowing us to conduct a greater number of simulations to efficiently validate our results. Considering those simulations for deposition result in an aerosol airborne time of approximately half a second, this interval is deemed satisfactory for the backward difference methods. To validate appropriate conversion of each numerical solution we run the simulations with decreasing time scale (see Figure S9a) and for each presented case we theoretically estimated the corresponding Global Error (see Figure S9b). A summary of all the global errors is supplied in Table S.


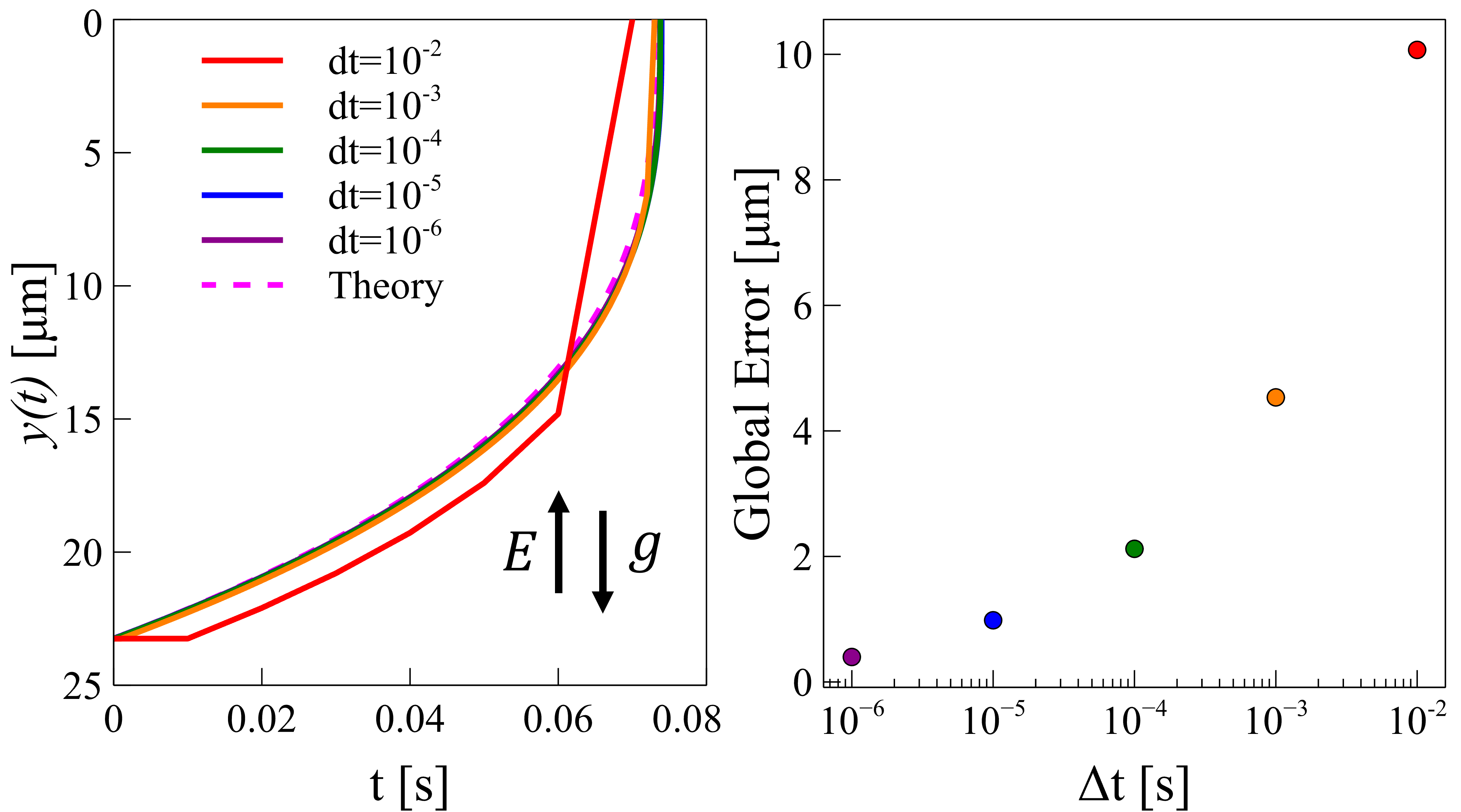


Figure S9: Numerical simulation convergence analysis for 0.2 $\mu m$ with q= 135e (a) Particle trajectories computed with decreasing time steps (colored solid lines), compared against the analytical solution (dashed line) from Equation 9 in the main paper. (b) Corresponding global error for each time step, shown in matching colors, demonstrating convergence of the numerical solution toward the analytical reference.

We used the Explicit Euler Method to solve newton 2^nd^ law for the particle trajectory in both y and x axis. The time interval chosen was $\Delta t$ =$0.1 \mathrm{ms}$ apart from q=415e case which deposited rapidly and hance $t=1 \mu m$. The explicit Euler Method truncated the Taylor series at the second order term and for that:

$$y\left( t+\Delta t \right)=y\left( t \right)+\Delta t\dot{y}\left( t \right)+\underset{e_{i} \sim O\left( \Delta t^{2} \right).}{\underbrace{\frac{\Delta t^{2}}{2}\ddot{y}\left( t \right)+\ldots}}$$

Neglecting terms of $O\left( \Delta t^{2} \right)$ and higher reduce the Taylor expansion to the Explicit Euler method. Hence, the local truncation error (LTE) of a single iteration is of $e_{i} \sim O\left( \Delta t^{2} \right).$ To determine the global truncation error $E_{n}$ over all $n$ steps of the simulation we assume the truncation error mainly depends on $\Delta t$:

$$E_{n}= \sum_{i}^{n} e_{i}= \sum_{i}^{n} \frac{\Delta t^{2}}{2}\ddot{y}\left( t \right)=\sum_{i}^{n} \frac{\Delta t^{2}}{2}a(t)$$

| $E_{n}=\sum_{i}^{n} \frac{\Delta t^{2}}{2}a(t)$ | **(**46) |
| --- | --- |

We can value this term through the simulation. An additional approach is to simplify and assume uniform acceleration:

$$E_{n}\approx\frac{\Delta t^{2}}{2}\ddot{y}\left( t \right)\sum_{i}^{n} 1=\frac{\Delta t^{2}}{2}\ddot{y}\left( t \right)*n$$

We recall that $n=\frac{t_{f}}{\Delta t}$, where $t_{f}$ is the overall simulation time.

$$E_{n}\approx\frac{\Delta tt_{f}}{2} \ddot{y}\left( t \right)$$

Considering the initial height $y_{0}$ and the total simulation time $t_{f}$ to estimate the second derivative as: $\frac{d^{2}y}{dt^{2}} \sim\frac{y_{0}}{t_{f}^{2}}$, in conclusion:

| $E_{n}\approx\frac{y_{0}}{2}\frac{\Delta t}{t_{f}}$ | **(**47) |
| --- | --- |

Let us calculate the approximation for the error, given that the initial height ($y_{0}=22.7\mu m)$ and interval time in all simulation are known:

| $d_{p} [ \mu m]$ | $q[e]$ | $t_{f} [s]$ | $\Delta t$ | $E_{n}\approx\frac{y_{0}\Delta t}{2t_{f}}$Error approximation [m] | $E_{n}=\sum_{i}^{n} \frac{\Delta t^{2}}{2}a_{i}$Real Simulation Error [m] |
| --- | --- | --- | --- | --- | --- |
| $0.2$ | 415 | 0.0076 | 1 $\mu s$ | $1.5\times{10}^{-9}$ | $7.66\times{10}^{-7}$ |
|  | 135 | 0.151 | 0.1 ms | $7.5\times{10}^{-9}$ | $2.25\times{10}^{-6}$ |
|  | 13 | 1.2 | 0.1 ms | $9.5\times{10}^{-10}$ | $2.48\times{10}^{-9}$ |
| $2$ | 650 | 0.195 | 0.1 ms | $5.8\times{10}^{-9}$ | $2.21\times{10}^{-6}$ |
|  | 65 | 0.481 | 0.1 ms | $2.4\times{10}^{-9}$ | $2.42\times{10}^{-9}$ |

Table S7: Global Error result both theoretical approximation and from the Numerical simulation

As shown, the actual simulation errors are in the order of no more than a few microns. Notably, in trivial cases ($q=13e, q=65e$) there is a good comparison with the two error approaches while all rest significant charged cases (dominant Inc), the deviation between simulation and the error estimation increases. We recall that near the tissue, due to the sharp acceleration caused by the electrostatic force, this increase was expected, given the inverse-square dependence of the force on distance ($F_{e}\sim y^{-2}$). Nevertheless, the simulations align closely with the analytical predictions presented in the paper, confirming that the observed discrepancies fall within the expected range and validating the accuracy of the numerical code.

# References

[1] R. Bessler, S. Bhardwaj, D. Malka, R. Fishler, and J. Sznitman, “Exploring the role of electrostatic deposition on inhaled aerosols in alveolated microchannels,” *Sci. Rep.*, vol. 13, no. 1, pp. 1–14, 2023, doi: 10.1038/s41598-023-49946-w.

[2] B. S. Cohen, J. Q. Xiong, C.-P. Fang, and W. Li, “Deposition of Charged Particles on Lung Airways,” *Health Phys.*, vol. 74, no. 5, 1998, [Online]. Available: https://journals.lww.com/health-physics/Fulltext/1998/05000/Deposition_of_Charged_Particles_on_Lung_Airways.2.aspx.

[3] R. Fishler, M. K. Mulligan, and J. Sznitman, “Acinus-on-a-chip: A microfluidic platform for pulmonary acinar flows,” *J. Biomech.*, vol. 46, no. 16, pp. 2817–2823, 2013, doi: 10.1016/j.jbiomech.2013.08.020.
